# ACHealthChain blockchain framework for access control and privacy preservation in healthcare

**DOI:** 10.1038/s41598-025-00757-1

**Published:** 2025-05-14

**Authors:** Ahmed M. Tawfik, Ayman Al-Ahwal, Adly S. Tag Eldien, Hala H. Zayed

**Affiliations:** 1 Computer Science Department, Faculty of Computers and Artificial Intelligence, Benha, Egypt; 2 Communication and Electronics Department, Pyramids-Institute for Engineering and Technology, Giza, Egypt; 3 Electrical Engineering Department, Faculty of Engineering at Shoubra, Benha, Egypt; 4 Faculty of Engineering, Egypt University of Informatics (EUI), Cairo, Egypt

**Keywords:** Blockchain, Security, Privacy, Electronic health records, Access control, Health care, Engineering

## Abstract

Ensuring privacy and confidentiality in healthcare data management remains a critical challenge. Traditional centralized access control mechanisms are susceptible to security breaches, including unauthorized access, data leakage, and single points of failure, as well as privacy violations such as patient record exposure and improper data sharing. To address these issues, this paper proposes ACHealthChain, a blockchain-based framework leveraging Hyperledger Fabric for decentralized and transparent access control. The framework integrates the InterPlanetary File System (IPFS) for decentralized storage and ensures privacy through Hyperledger Fabric channels. ACHealthChain features PolicyChain for fine-grained access control and revocation, structuring patient health data into separate subchains for EHRs and diagnoses with permissioned access. Additionally, LogChain enhances auditing and accountability. A series of experiments evaluate ACHealthChain’s performance and scalability, considering metrics such as throughput, latency, and resource utilization. Results demonstrate that ACHealthChain improves throughput by 19.7% and reduces latency by 87%, outperforming existing frameworks built on the same platform. The scalability analysis further confirms the framework’s capability to handle increasing workloads within an expanding blockchain network. ACHealthChain presents a promising solution for secure and efficient healthcare data sharing with potential real-world applications.

## Introduction

The healthcare industry has rapidly embraced digital technologies, with Electronic Health Records (EHRs) becoming the standard for storing and managing patient information^1^. EHRs improve accessibility, streamline workflows, and facilitate data-driven research^2,3^. However, these advancements raise critical privacy and security concerns^4^. Unauthorized access or breaches can lead to identity theft, insurance fraud, and compromised healthcare decisions, making robust security measures essential. Traditional access control models, such as role-based access control (RBAC)^5^, regulate data access through predefined roles and privileges. While effective, these models struggle to meet the evolving needs of dynamic healthcare environments. Healthcare professionals require fine-grained access control, ensuring authorized individuals can access specific parts of a patient’s records. Additionally, auditing and compliance enforcement are challenging with conventional models.

Blockchain technology^6,7^ has emerged as a promising solution for secure and privacy-preserving access control in healthcare. Originally developed for cryptocurrencies like Bitcoin^8^, blockchain is now widely applied across various domains due to its key properties. Its decentralized nature ensures data integrity and availability, eliminating reliance on a central authority. Consensus mechanisms validate transactions, while transparency^9^ enables auditable records, enhancing accountability. Additionally, immutability^10^ prevents unauthorized modifications, ensuring trust in patient records. Smart contracts automate fine-grained access control, ensuring only authorized individuals can access or modify healthcare data.

Storing complete patient EHRs directly on a blockchain is impractical due to scalability and privacy concerns. Instead, the InterPlanetary File System (IPFS)^11^ provides a decentralized solution for managing large medical files, using cryptographic hashes to ensure data integrity and efficient retrieval. Integrating blockchain-based access control with IPFS allows secure storage and management of encrypted EHR data while maintaining transparency and scalability.

Popular blockchain platforms, such as Ethereum^12^ and Hyperledger Fabric^13^, offer different advantages based on decentralization, privacy, and performance needs. This paper employs the Hyperledger Fabric as a permissioned blockchain framework for providing features like modular architecture, flexible consensus protocols, and privacy through channels. Hyperledger Fabric^13^ is often used in various industries such as the healthcare industry, where privacy, scalability, and fine-grained access control are essential.

In this paper, we introduce ACHealthChain, a blockchain-based framework designed to enhance access control and privacy preservation in healthcare data management. Unlike traditional approaches that rely on multiple channels in Hyperledger Fabric, ACHealthChain leverages multiple chaincodes to reduce communication overhead and achieve modularity within the same blockchain network. This approach allows for separate development and control of smart contracts, ensuring flexibility, improved isolation, and tailored access control policies for different stakeholders, such as patients and healthcare providers. By implementing distinct chaincodes, the framework enables fine-grained access control, ensuring that patients retain full authority to grant or revoke permissions over their EHRs, thereby enhancing privacy and security. The major contributions of this work are summarized as follows:

1. **Novel blockchain-based access control framework:** We propose ACHealthChain, which introduces a dedicated PolicyChain to facilitate fine-grained access control and revocation mechanisms. The framework separates patient healthcare data into EHRs and diagnoses, distributing them across different permissioned subchains for improved data management and security.
2. **Enhanced auditability and accountability:** ACHealthChain integrates a LogChain mechanism to ensure transparent tracking of data access and usage, providing robust auditing capabilities crucial for regulatory compliance and security.
3. **Implementation and performance evaluation:** The framework is implemented on Hyperledger Fabric, utilizing the Hyperledger Caliper tool for comprehensive evaluation. This combination allows for an accurate, realistic assessment of the framework’s performance and enables meaningful comparisons with other frameworks.
4. **Scalability and performance superiority:** Experimental results demonstrate that ACHealthChain achieves high efficiency with superior throughput and low latency compared to existing frameworks built on the same platform. The scalability analysis confirms its capability to handle increasing workloads in real-world healthcare settings.

The rest of this paper is organized as follows: section “Background and related work” provides background information and discusses related work in the field of blockchain-based access control and privacy preservation in healthcare. Section “ACHealthChain: proposed framework” presents the proposed framework, providing a detailed description of its key components and functionalities. Section “ACHealthChain: implementation and scenarios” discusses the proposed framework implementation, including scenarios related to patient, healthcare provider manipulation, and healthcare data access. Section “Security and privacy analysis” discusses the security and privacy aspects of the proposed framework. Section “Evaluation and experimental results” presents the evaluation and experimental results obtained from the framework. Finally, Section “Conclusion” concludes the paper by summarizing the contributions, discussing limitations, and providing directions for future research.

## Background and related work

This section effectively provides the necessary background information and presents the relevant work of blockchain based access control frameworks in the healthcare sector. It offers a brief overview of each framework, highlighting their key features and limitations.

### Background

Blockchain technology^14^ is a decentralized and distributed ledger technology that enables secure and transparent recording of transactions and data. Initially introduced as the underlying technology for cryptocurrencies like Bitcoin^8^, blockchain has since expanded its applications to various industries beyond finance. Platforms such as Ethereum^12^, Hyperledger Fabric^13^, and others have further developed blockchain technology, offering more flexible and customizable solutions for specific use cases. At its core, a blockchain is a growing chain of blocks, each containing a list of transactions or data. These blocks are linked using cryptographic hashes, forming an immutable and tamper-resistant record of all stored transactions or data.

Blockchain technology’s decentralized nature eliminates reliance on central authorities or intermediaries, operating instead on a network of nodes that collectively validate transactions. Consensus protocols, such as Proof of Work (PoW)^8^, Proof of Stake (PoS)^15^, and Practical Byzantine Fault Tolerance (PBFT)^16^, ensure transaction validity and maintain blockchain integrity by enabling nodes to agree on the blockchain’s state.

Transparency and immutability form the foundational aspects of blockchain technology^9,10^. Transparency ensures that all participants in the network can independently verify and validate recorded transactions or data entries, fostering trust and accountability. Immutability guarantees that once a transaction or data is added to the blockchain, it cannot be altered or deleted without detection. Each block contains a reference to the previous block, making tampering highly impractical as any changes to previous blocks would require re-computing the cryptographic hashes of subsequent blocks.

Blockchain technology offers enhanced security due to its decentralized nature, which makes it more resilient to attacks and data breaches. Additionally, its transparency and immutability enable easy auditability and traceability of assets or data. However, these features also pose challenges in terms of privacy protection, as sensitive information is exposed to all network participants. Various approaches, such as using permissioned or private blockchains, restrict access to a predefined set of participants, allowing for better control over data access and enhancing privacy.

Advancements in cryptography have led to the development of privacy-enhancing technologies for blockchain systems. Techniques like Secure Multiparty Computation (SMPC)^17^ and homomorphic encryption^18^ enable secure computation and verification without revealing sensitive data. For secure data storage, blockchain-based solutions have emerged that combine on-chain verification with off-chain storage^19^. This hybrid approach typically utilizes encrypted databases or decentralized file systems like IPFS^11^ to store sensitive data separately from the public ledger while maintaining cryptographic links to the blockchain. Merkle trees^20^ and other authenticated data structures provide integrity proofs for this off-chain data. Furthermore, selective disclosure mechanisms, implemented through smart contracts and zero-knowledge techniques^21^, allow users to reveal only necessary information while preserving overall data privacy. In recent years, various proposals for blockchain-based access control frameworks have emerged to enhance healthcare privacy, as discussed in the following sub-section.

### Related work

This section reviews relevant work, outlining key features and limitations of each framework. A summary is presented in Table 1.

MedRec^22^ introduces blockchain-based EHRs management, overcoming traditional storage limitations with smart contracts. It employs three key contracts: Register Contract (RC) for privacy, Patient-Provider Relationship Contract (PPR) for data storage, and Summary Contract (SC) for record tracking. While ensuring secure data storage, MedRec lacks fine-grained access control enforcement.

**Table 1:** A qualitative comparison of various blockchain-based frameworks for managing access control in healthcare environments.

Framework	Year	Blockchain type	Consensus protocol	Blockchain platform	Throughput	Latency	Storage overhead	Limitations of the framework
MedRec^22^	2016	Public	PoW	Ethereum	Low	High	Medium	No fine-grained access control
MedShare^23^	2017	Private	Proposed by authors	Proposed by authors	Low	High	Medium	Centralized components
$\hbox {MediChain}^{TM}$ ^24^	2018	Private	PBFT	Hyperledger fabric	High	Low	Medium	No patient-centric revocation
Healthchain^25^	2019	Public, Consortium	PoW and PBFT	Proposed by authors	Medium	Medium	Medium	PoW and RSA computational overhead
SPChain^26^	2021	Public	PoW and BFT	Bitcoin (Simulation)	Low	High	Medium	Bitcoin scalability limitations
BCHealth^27^	2021	Private	PoA	Proposed by authors	Medium	Medium	High	Storage overhead for dual chains
Xiang and Zhao^28^	2022	Private, Consortium	Proposed by authors	Proposed by authors	High	Low	Medium	Depends on CSP, affecting decentralization.
Díaz et al.^29^	2023	Private	PBFT	Hyperledger fabric	Medium	Medium	Medium	Limited storage scalability. External database reliance.
Lax et al.^30^	2024	Public	PoS	Ethereum	Medium	Medium	High	Unverified implementation. High computation overhead.
ACHealthChain	2025	Consortium	PBFT	Hyperledger fabric	High	Low	Low	Limited interoperability with public blockchain- based healthcare solutions.

The qualitative metrics are classified as follows-Throughput (High: > 300 TPS, Medium: 100–300 TPS, Low: < 100 TPS), Latency (Low: < 100 ms, Medium: 100–500 ms, High: > 500 ms), and Storage Overhead (Low: < 500 MB, Medium: 500 MB-2 GB, High: > 2 GB)-based on common blockchain performance benchmarks and reported experimental results in the cited frameworks

MeDShare^23^ employs a permissioned blockchain approach to enable patients to manage and monitor their EHRs through smart contracts. It operates through three layers: a data layer for user classification, a data query layer for access management, and a structural layer for authentication and data verification. Requests are verified by an authenticator, and smart contracts enforce policy compliance. MeDShare distinguishes between high-sensitive and low-sensitive data, aiming to minimize breach reports for the latter. However, its performance evaluation focuses mainly on network latency, potentially limiting a holistic assessment.

$\hbox {MediChain}^{TM}$^24^ is a permissioned blockchain framework built on Hyperledger^13^, aiming to provide data access control. Original EHRs reside in cloud storage, while their hashes are stored on the blockchain. $\hbox {MediChain}^{TM}$ allows data owners to create smart contracts for access control, known as Discretionary Access Control (DAC). The framework employs the PBFT consensus protocol^16^ for blockchain integrity. However, $\hbox {MediChain}^{TM}$ lacks features for essential access control aspects like delegation and revocation.

Healthchain^25^ is a hybrid blockchain framework addressing privacy and storage costs for large-scale EHRs. It utilizes IPFS^11^ for efficient storage while preserving patient privacy on the public blockchain network. It supports fine-grained access control, enabling patients to revoke access dynamically by updating their keys on the blockchain. However, its access control is restricted to predefined roles, and it lacks flexible policy definition. Processing times are relatively high due to RSA cryptography, despite its robust security features.

SPChain^26^ is a blockchain framework designed for secure medical data transfer and privacy preservation in e-health systems. It employs keyblock and microblock as blockchains for fast retrieval of patients’ EHRs. Keyblocks store register transactions, while microblocks link label and medical transactions to individual patients. SPChain ensures patient privacy using proxy re-encryption techniques and chameleon hash functions^31^ to store medical history securely. The system employs three transaction types: register, label, and medical. It combines PoW^10^ and Byzantine Fault Tolerance (BFT)^32^ for consensus. While offering advantages like low storage overhead and robust security, SPChain should improve communication efficiency and throughput for enhanced performance.

BCHealth^27^ is a blockchain-based architecture allowing data owners to set access permissions for EHRs. Patient data isn’t shared on the blockchain without patient consent. It comprises two chains: the data chain storing EHRs and the access control chain managing access rules. A clustering technique enhances scalability and capacity, enabling effective data and transaction management. BCHealth can promptly alert physicians about disease symptoms, enabling timely action. It employs the Proof-of-Authority (PoA)^33^ consensus protocol for efficiency and security. While it offers efficient computation and processing, retrieving health information may experience slight delays, and storage overhead could be higher than centralized solutions. Optimizing node count and clustering arrangement is essential for improved performance and resource utilization in BCHealth.

The blockchain-assisted SABE framework^28^ enhances e-health access control using Attribute-Based Encryption (ABE)^34^. It enables keyword-based searches and ensures fairness, security, and efficiency through blockchain integration. Each hospital has a private blockchain, while a consortium blockchain stores EHR keywords. Access records are audited on the blockchain, and encrypted EHRs are stored in the cloud for quick and lightweight decryption. This approach prioritizes attribute privacy and improves the efficiency of Cloud Service Provider (CSP) searches, offering cost-effective solutions. However, decentralization may be compromised depending on the CSP.

Díaz et al.^29^ present a dual-channel blockchain framework utilizing Hyperledger Fabric for scalable EHR management in healthcare systems. This system is structured around a decentralized network of hospitals, labs, research centers, and insurance agencies, where each organization manages user access to sensitive health data via distinct roles. Employing the RAFT consensus mechanism, the model uses separate channels to optimize scalability and data privacy, allowing unique data access and permissions per channel. While the dual-channel model provides reduced latency and improved throughput, further refinement is needed for enhanced interoperability and storage efficiency.

Lax et al.^30^ proposed a blockchain-based framework that decouples patient identities from their e-health records while restricting access to authorized entities. The system utilizes digital identities for access control and is built on the Ethereum blockchain. However, the requirement for patient authentication during every EHR operation may hinder usability in clinical settings. Additionally, using a public blockchain with the PoS consensus protocol^15^ introduces inherent latency that could impact time-sensitive healthcare workflows. The authors note that the framework remains a theoretical proposal without full implementation.

Our proposed framework, ACHealthChain, addresses key limitations found in existing blockchain-based access control frameworks, as outlined in Table 1. Specifically, these solutions face challenges such as the lack of fine-grained access control, high computational overhead, scalability constraints, and dependence on centralized components or external storage solutions. Notably, MedRec lacks fine-grained access control, while frameworks such as MediChain^TM^ and Díaz et al. exhibit storage scalability limitations. Additionally, solutions built on public blockchains, such as SPChain and Lax et al., suffer from high computational overhead and latency due to consensus mechanisms like PoW and PoS. In contrast, ACHealthChain leverages Hyperledger Fabric with PBFT consensus to achieve high throughput and low latency while maintaining a decentralized, permissioned environment that supports structured data management. Furthermore, ACHealthChain minimizes storage overhead by integrating IPFS, enhancing data availability and privacy. This combination ensures that patient data remains secure and accessible only to authorized entities while enabling efficient policy-based access control with revocation capabilities.

## ACHealthChain: proposed framework

**Fig. 1:**
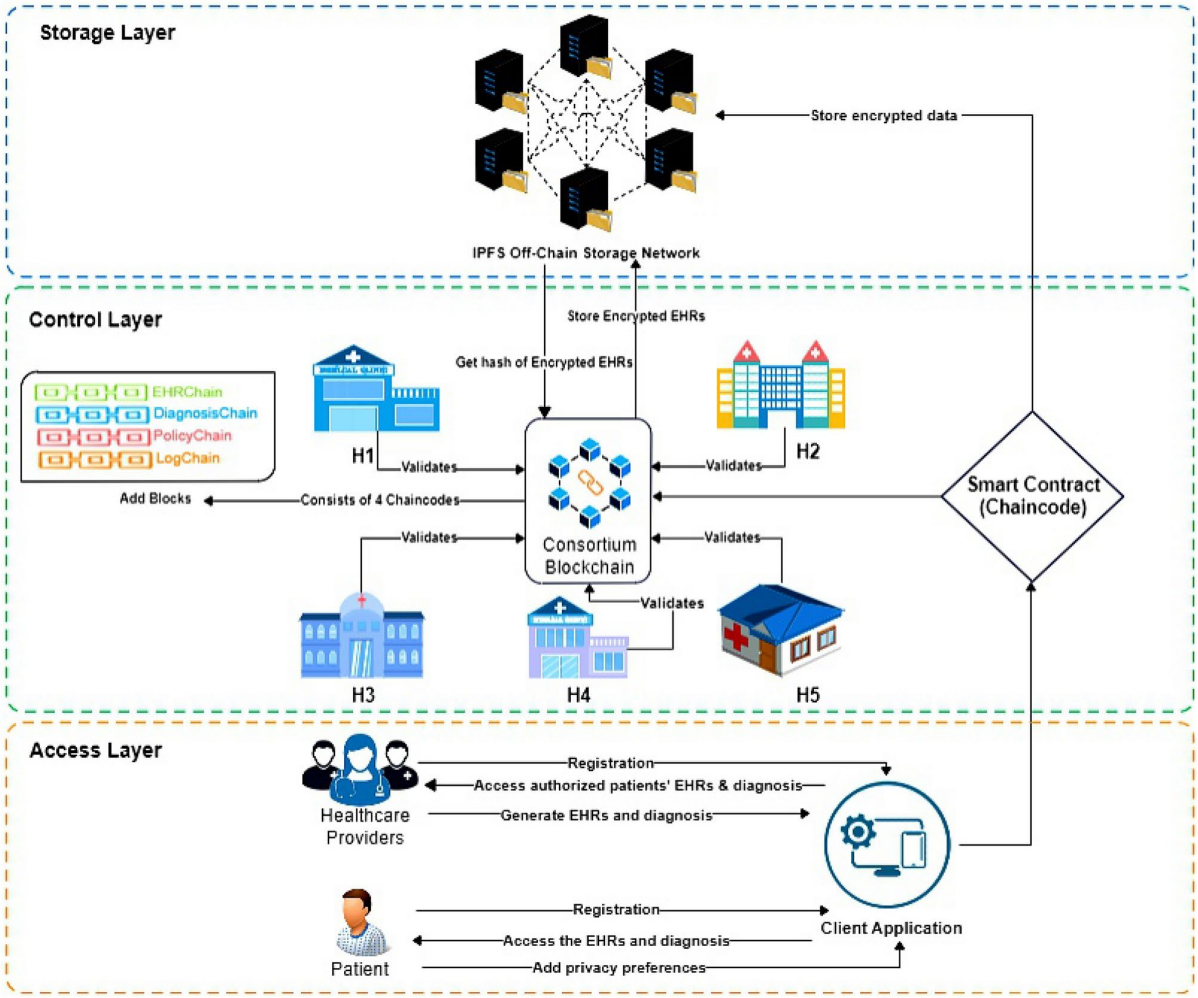
ACHealthChain: the proposed framework architecture.

We propose the ACHealthChain framework, which leverages blockchain technology to establish secure and decentralized access control management in the healthcare domain. The framework is implemented on Hyperledger Fabric, a permissioned blockchain platform^13^, and is structured into three layers: the storage layer, the control layer, and the access layer-each serving a distinct role in ensuring secure and decentralized access control, as depicted in Fig. 1. The Storage Layer forms the foundation of the architecture, ensuring the secure and immutable storage of healthcare data.

### Storage layer

Instead of utilizing Blockchain for healthcare data storage, the proposed framework utilizes the IPFS distributed storage system, which offers several benefits when used in conjunction with blockchain technology^11^:

1. Decentralized storage: IPFS utilizes a distributed network of nodes for decentralized storage, increasing data availability and resistance to censorship or single points of failure.
2. Immutable content addressing: IPFS employs content-based addressing, where files are identified and retrieved based on their content, ensuring secure and immutable storage. This feature is especially valuable for maintaining data integrity in blockchain applications.
3. Reduced redundancy: IPFS employs data deduplication, storing files with identical content only once. This reduces redundancy and optimizes storage space.
4. Faster data retrieval: IPFS uses a distributed hash table to locate and retrieve files. It accelerates data retrieval by automatically searching for the nearest nodes with the file, resulting in faster and more efficient data retrieval compared to centralized storage systems.
5. Data integrity and authenticity: IPFS ensures data integrity by utilizing cryptographic hashing. The unique hash of each file identifies its content, preventing tampering or unauthorized modifications without detection.

For privacy protection, healthcare data is encrypted using the Advanced Encryption Standard (AES)^35^ before being stored in IPFS storage. This choice of AES is preferred over other encryption schemes, such as ABE^34^, which is renowned for its complex fine-grained access control capabilities. AES is chosen due to its widespread adoption and superior performance compared to ABE in handling large volumes of data, making it particularly suitable for securing EHRs^36^. This decision is especially relevant given that our framework utilizes a custom blockchain called PolicyChain for fine-grained access control.

While the Storage Layer ensures secure and decentralized data storage, managing access control policies and enforcing security mechanisms requires a higher-level framework. This is where the Control Layer comes into play. It governs the operational logic of ACHealthChain by defining policies, enforcing data access rules, and ensuring transaction integrity through smart contracts and consensus mechanisms, which collectively regulate interactions between stakeholders while maintaining data security and privacy.

### Control layer

The control layer governs the ACHealthChain framework by enforcing access policies and ensuring proper data flow between network participants. It comprises smart contracts (chaincode), modular subchains, and a consensus protocol.

#### Smart contracts (Chaincode)

In ACHealthChain, smart contracts-referred to as chaincode-encode the business logic governing transactions. Written in Java, chaincode executes on endorsing peers, validating transactions against predefined policies before appending them to the blockchain. Chaincode ensures automation, security, and compliance with access control rules.

#### Subchains and their role

In the proposed ACHealthChain framework, we aim to achieve modularity within the blockchain network by implementing different chaincodes rather than creating separate channels, which would result in increased computational and communication overhead. Each chaincode represents a unique set of rules and functions, encapsulating specific business logic. The isolation of chaincode logic facilitates easier maintenance and debugging, leading to improved overall network efficiency and minimizing the impact of changes to one part of the system on other parts, thereby enhancing system flexibility. These chaincodes include EHRChain for secure sharing of EHRs between patients and healthcare providers, DiagnosisChain for sharing doctors’ diagnoses between patients and healthcare providers, PolicyChain for determining healthcare data access policies based on patient preferences, and LogChain for handling access logs data for both patients and healthcare providers, ensuring proper auditing and tracking of data access.

The transactions in **EHRChain** consist of the following tuples: *EHRChainTx* <*Patient_ID, Timestamp, IPFS_EHR Address, Patient Signature, EHRChainTx ID>*

- *Patient_ID:* the public key of the patient who initiates the transaction.
- *Timestamp:* the transaction creation datetime is recorded in milliseconds.
- *IPFS_EHR Address:* the hash address of the encrypted EHR is stored in IPFS storage.
- *Patient Signature:* the encryption result is derived from the patient’s private key.
- *EHRChainTx ID:* the transaction’s identity is established by generating a hash of all its components, facilitating efficient retrieval and ensuring integrity.

Transactions within **DiagnosisChain** consist of the following components: *DiagnosisChainTx *<*Healthcare Provider ID, Timestamp, patient_ID, IPFS_Diagnosis Address, healthcare provider Signature, DiagnosisChainTx ID>*

- *Healthcare Provider ID:* the public key of the healthcare provider who initiates the transaction.
- *Timestamp:* the transaction creation datetime is recorded in milliseconds.
- *Patient_ID:* the patient’s public key, which is used to address them in the sub-chain of the EHRChain.
- *IPFS_Diagnosis Address:* the hash address of the encrypted diagnosis is stored in IPFS storage.
- *healthcare provider Signature:* the encryption result is derived from the healthcare provider’s private key.
- *DiagnosisChainTx ID:* the transaction’s identity is established by generating a hash of all its components, facilitating efficient retrieval and ensuring integrity.

The transactions within **PolicyChain** consist of the following components: *PolicyChainTx *<*Granter_ID, Grantee_ID, Timestamp, EHR_Type, IPFS_hash Address, enveloped symmetric_key, Start_Date, End_Date, isValidPolicy, Granter Signature, PolicyChainTx ID>*. The granter or grantee can be a patient or healthcare provider.

- *Granter_ID:* the public key of the granter who initiates the transaction.
- *Grantee_ID:* the public key of the grantee who is granted access to the healthcare data.
- *Timestamp:* the transaction creation date time is recorded in milliseconds.
- *EHR_Type:* the type of healthcare data that the authorized requester can access, e.g., ECG, EEG data.
- *IPFS_hash Address:* the hash address of the encrypted healthcare data is stored in IPFS storage.
- *Enveloped_Symmetric_Key:* the granter’s symmetric key is encrypted using the grantee’s ID (public key) in order to decrypt the returned healthcare data from the IPFS.
- *Start_Date:* the start date of the granter’s policy.
- *End_Date:* the expiration date of the granter’s policy.
- *isValidPolicy:* this is a boolean value that indicates the validity of the policy. It is set to TRUE for valid policies of accessing EHRs and FALSE for revoking access permission.
- *Granter Signature:* the encryption result is derived from the granter’s private key.
- *PolicyChainTx ID:* the transaction’s identity is established by generating a hash of all its components, facilitating efficient retrieval and ensuring integrity.

The transactions within **LogChain** consist of the following components: *LogChainTX *<*Requester_ID, Timestamp, IPFS_EHR Address, Access_Logs, Requester Signature, LogChainTx ID>*

- *Requester_ID:* the public key of the Requester who initiates the transaction.
- *Timestamp:* the transaction creation datetime is recorded in milliseconds.
- *IPFS_EHR Address:* the hash address of the encrypted EHR is stored in IPFS storage.
- *Access_Logs:* The information includes a summary of the types of accessed EHRs, the individuals who accessed them, and other relevant details.
- *Requester Signature:* the encryption result is derived from the Requester’s private key.
- *LogChainTx ID:* the transaction’s identity is established by generating a hash of all its components, facilitating efficient retrieval and ensuring integrity.

#### Consensus protocol

The consensus protocol is a crucial mechanism in distributed systems, including blockchain networks, to establish agreement and consensus among multiple nodes regarding the validity and order of transactions or events. Its purpose is to ensure that all participants in the network reach a consistent state and agree on state changes. The ACHealthChain framework employs the PBFT consensus protocol^16^ alongside the Raft ordering service^37^ to ensure fault tolerance and consensus across nodes in the distributed network. The Raft enables leader election, log replication, and commit operations to ensure fault tolerance and consistency. Compared to Solo and Kafka ordering services, Raft offers advantages in terms of fault tolerance, log replication, and simplicity. Its robustness and reliability make it a suitable choice for achieving consensus in the ACHealthChain framework.

With a well-structured Control Layer governing transactions, policies, and security rules, it is essential to have a mechanism that ensures only authorized entities can interact with the system. The Access Layer serves this purpose by handling authentication, user registration, and identity management. This layer is responsible for verifying the credentials of participants, such as patients and healthcare providers, before they can interact with the blockchain network. By implementing robust authentication mechanisms and role-based access controls, the Access Layer reinforces the security and usability of the ACHealthChain framework.

### Access layer

The access layer in the ACHealthChain framework controls access permissions in the blockchain network, allowing only authenticated participants to interact with the system. It handles the registration and authentication processes for client nodes, prioritizing security and implementing effective authentication mechanisms.

#### Registration

The registration module in the proposed framework handles identity registration and verification processes. It securely stores certificates and generates new certificates for verified participants. It validates participant identities using X.509 certificates^38^ and utilizes a web service API for secure client access. In Hyperledger Fabric^13^, the Membership Service Provider (MSP) is responsible for managing participant identities and enforcing access control policies. The MSP issues digital certificates, maintains a Certificate Authority (CA), and ensures that only authorized participants can access the network.

#### Client nodes

In the proposed framework, the client nodes refer to the nodes that are used by patients and healthcare providers to interact with the healthcare blockchain network. These client nodes act as the endpoints for the users, allowing them to access and utilize the functionalities provided by the blockchain system.

1. **Patients node:** In this framework, each patient node has the responsibility of accessing and managing its EHRs, as well as setting privacy preferences to control who can access their healthcare data. Patient nodes employ Elliptic Curve asymmetric encryption, using their private key and the public key of the corresponding peer, to encrypt the data before sending it to the IPFS storage node. These nodes possess the capability to access and publish transactions, validate and commit new blocks of transactions received from consensus nodes to their local blockchain copy. Moreover, all patient nodes are capable of executing smart contracts.
2. **Healthcare providers node:** Healthcare provider nodes play a crucial role in the ACHealthChain framework by offering diagnoses based on healthcare data for specific patients and securely transmitting encrypted EHRs to the IPFS storage node. These EHRs may include diagnoses, medical laboratory reports, insurance documents, and other relevant information. Similar to patient nodes, healthcare provider nodes possess the same capabilities for blockchain operations, allowing them to access and publish transactions, validate and commit new blocks of transactions, and execute smart contracts.

With a solid understanding of the ACHealthChain framework in place, we now turn our attention to its implementation and explore various scenarios where it can be effectively applied. The use cases and scenarios where ACHealthChain demonstrates its potential to revolutionize access control in the healthcare industry.

## ACHealthChain: implementation and scenarios

This section provides a detailed explanation of the implementation of the primary chaincodes utilized in the ACHealthChain framework. To facilitate interaction with the Blockchain network, we employ a web service API that grants secure access to clients. The ACHealthChain framework prioritizes security by incorporating authentication and verification processes through the MSP. MSP is an essential component of the Hyperledger Fabric framework^13^. The MSP acts as a trusted authority within the network and is responsible for issuing digital certificates to network participants. These certificates are used for identity authentication and are crucial for establishing trust within the network. The MSP maintains a public key infrastructure (PKI) that includes the CA to issue and sign certificates. By utilizing the MSP, the Hyperledger Fabric framework ensures that only authorized participants can access and interact with the network. The initial step involves enrolling the hospital’s administrator through the MSP and importing their identity into the wallet. This identity comprises essential components such as MSP ID, identity version, certificate type, private key, and a public certificate. Once enrolled, the administrator gains the ability to register and enroll new users. The system restricts user roles to patients or healthcare providers in accordance with the proposed framework.

The ACHealthChain framework is designed with four distinct subchains, each serving a specialized function to enhance security, privacy, and access control in managing EHRs:

- **EHRChain:** This subchain is responsible for securely storing and managing patients’ EHRs. It ensures that healthcare providers can access patients’ health records while restricting access to other providers’ diagnoses, thereby maintaining data separation and privacy.
- **DiagnosisChain:** This subchain is dedicated to storing and organizing patient diagnoses separately from EHRs. By maintaining a distinction between health records and medical assessments, the framework upholds data modularity and prevents unauthorized inference attacks.
- **PolicyChain:** Serving as the core access control mechanism, the PolicyChain governs access permissions to both EHRs and diagnoses. It also facilitates the secure exchange of encrypted healthcare data stored on IPFS by providing the necessary enveloped symmetric key for decryption, ensuring that only authorized entities can access sensitive information.
- **LogChain:** To enforce accountability and traceability, the LogChain records detailed logs of both authorized and unauthorized access attempts. This mechanism strengthens the framework’s security by enabling auditing and monitoring of all interactions within the system.

To demonstrate the practical application of the ACHealthChain framework, we outline three key operational scenarios. These scenarios are accompanied by sequence diagrams and algorithms to provide a structured and clear illustration of system behavior:

- **Patient scenario:** In this scenario, patients store their EHRs in the EHRChain and define their privacy preferences. These preferences are transformed into access policies stored in the PolicyChain, ensuring that only authorized healthcare providers can access their health records.
- **Healthcare providers scenario:** Healthcare providers add diagnoses to the DiagnosisChain based on the retrieved patients’ EHRs (via the Healthcare Data Access Scenario). Additionally, access control policies are updated to define which entities can view the diagnoses, ensuring secure and structured information management.
- **Healthcare data access scenario:** This scenario governs the retrieval of patient EHRs and diagnoses from the EHRChain and DiagnosisChain, respectively. Access control policies stored in the PolicyChain determine authorization for healthcare providers or patients to access specific records. Furthermore, all access attempts-both authorized and unauthorized-are logged in the LogChain for security and auditing purposes.

This structured approach ensures a logical and secure workflow. For instance, when a healthcare provider needs to add a diagnosis, they must first retrieve the relevant EHRs through the Healthcare Data Access Scenario. Once the necessary health records are obtained, they proceed with the Healthcare Providers Scenario to record their diagnosis. This modular and layered design enhances security, privacy, and efficiency in managing sensitive healthcare data. The following subsections provide an in-depth exploration of each scenario, detailing the sequence of operations and the interaction between different subchains in ACHealthChain.

### Patient scenario

**Fig. 2:**
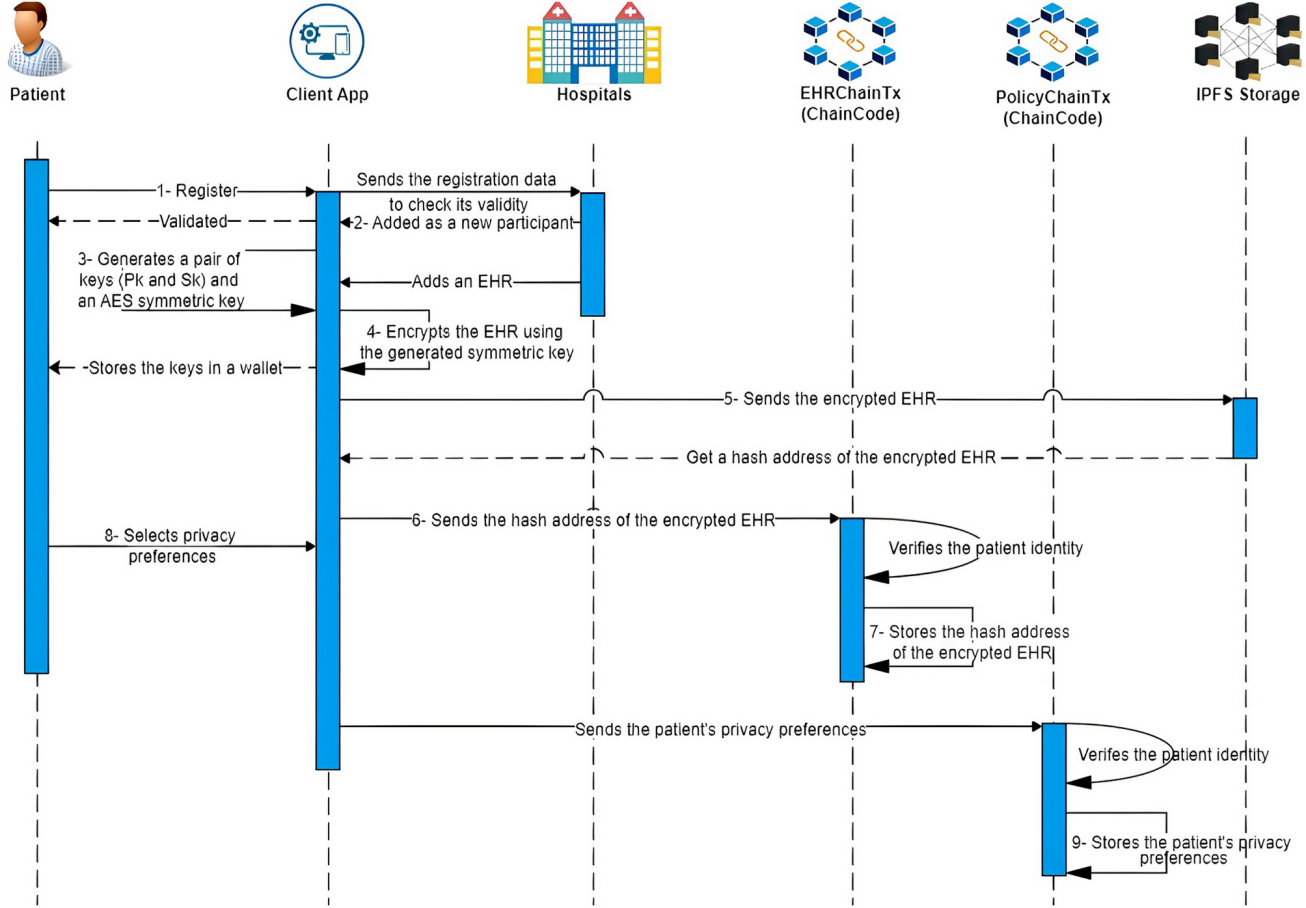
Patient scenario.

Patients can interact with their EHRs and customize privacy preferences, selecting specific healthcare providers authorized to access and manage their records. This empowers patients by granting them control over who can view and handle their sensitive health information, as detailed in Algorithm 1. The patient scenario is illustrated in Fig. 2 and follows these steps:

1. The patient submits identity-related information to initiate a registration request.
2. Upon successful identity validation, the patient is registered as a participant in the hospital network. A key pair (public key Pk and private key Sk) is generated and securely stored in the patient’s wallet.
3. The client app generates an AES symmetric key to encrypt the patient’s EHR, storing it securely in the patient’s wallet.
4. The participating hospital encrypts the newly added EHR using the client app.
5. The encrypted EHR is transmitted to an IPFS storage node, and its corresponding hash address is retrieved.
6. The client app sends the EHR’s hash address to the blockchain network.
7. The patient’s identity is verified via chaincode within the permissioned blockchain (EHRChain). Once verified, the hash address of the patient’s EHR is securely stored within EHRChain.
8. The patient sets privacy preferences, specifying authorized healthcare providers. These preferences are then sent to the blockchain network.
9. The patient’s identity is verified using PolicyChain chaincode within the permissioned blockchain. Upon successful verification, the privacy preferences are securely stored in PolicyChain as transactions. These transactions contain details such as the patient’s public key, the authorized healthcare provider’s public key, timestamp, IPFS address of the EHR, EHR type, an encrypted symmetric key (enveloped with the provider’s public key), access duration, policy validation, and the patient’s signature.

#### EHRs access revocation

If a patient decides to revoke a specific grantee’s permission to access the EHRs, he/she creates a policy transaction with “isValidPolicy = false” on the blockchain. When the requester attempts to access the EHRs, the PolicyChain’s chaincode first verifies the validation parameter “isValidPolicy” in the last policy transaction relevant to the patient and healthcare provider. If the validation is false, the healthcare provider will be denied access to the EHRs, as detailed in Algorithm 2.

**Algorithm 1:**
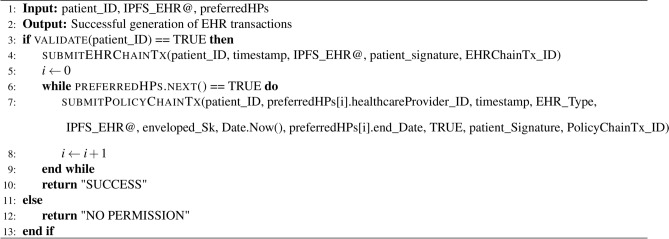
Chaincode of generating patient EHRs

**Algorithm 2:**
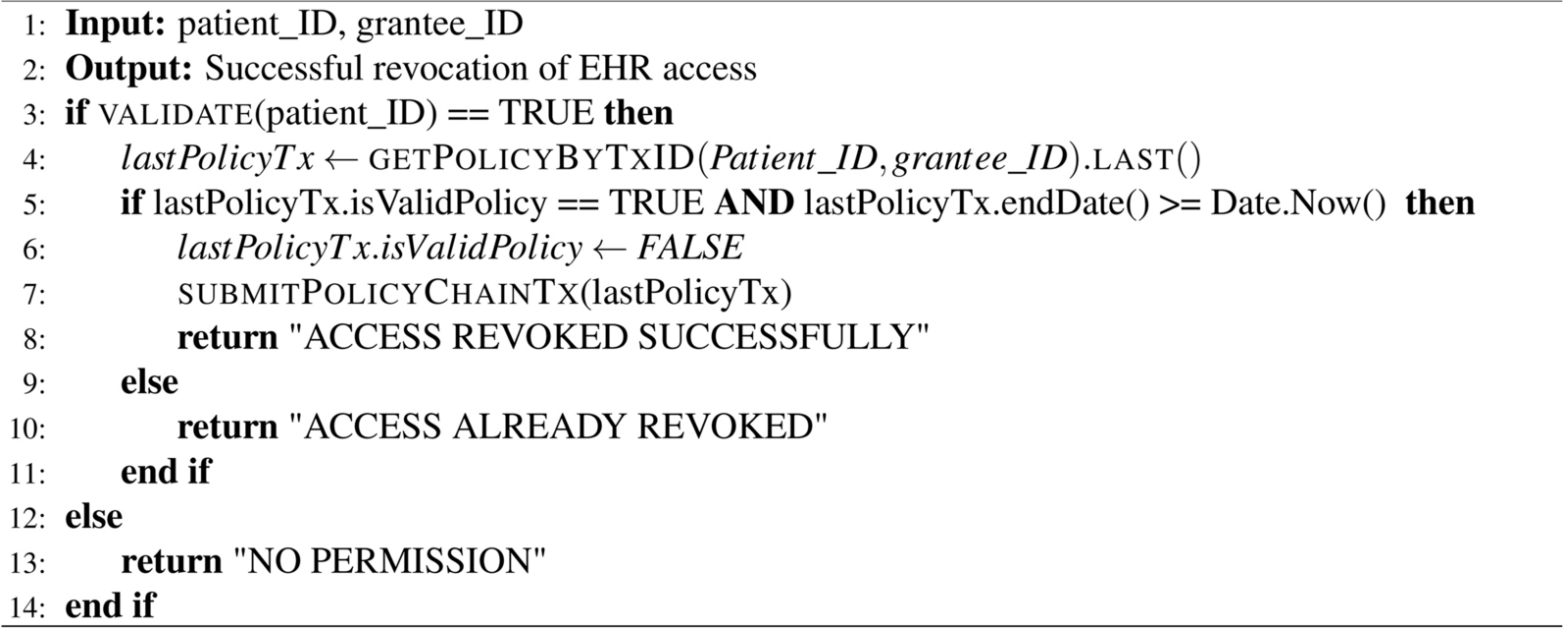
Chaincode of revoking access to EHRs

**Algorithm 3:**
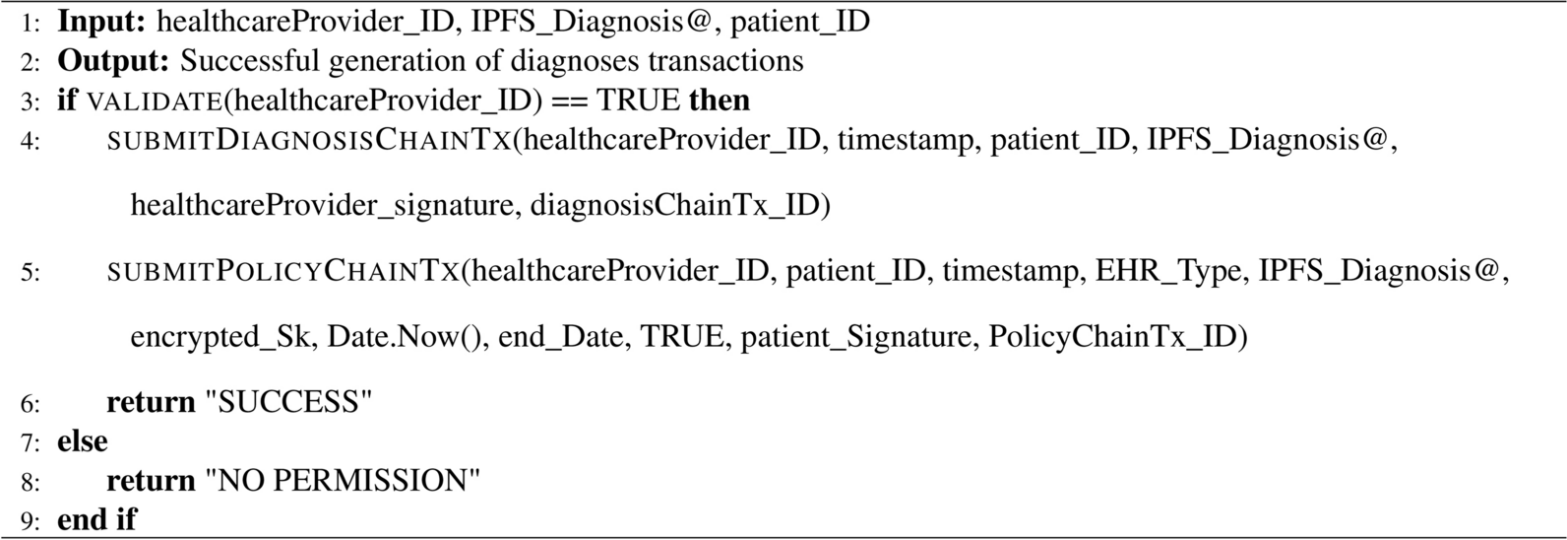
Chaincode of generating patient diagnosis

### Healthcare providers scenario

**Fig. 3:**
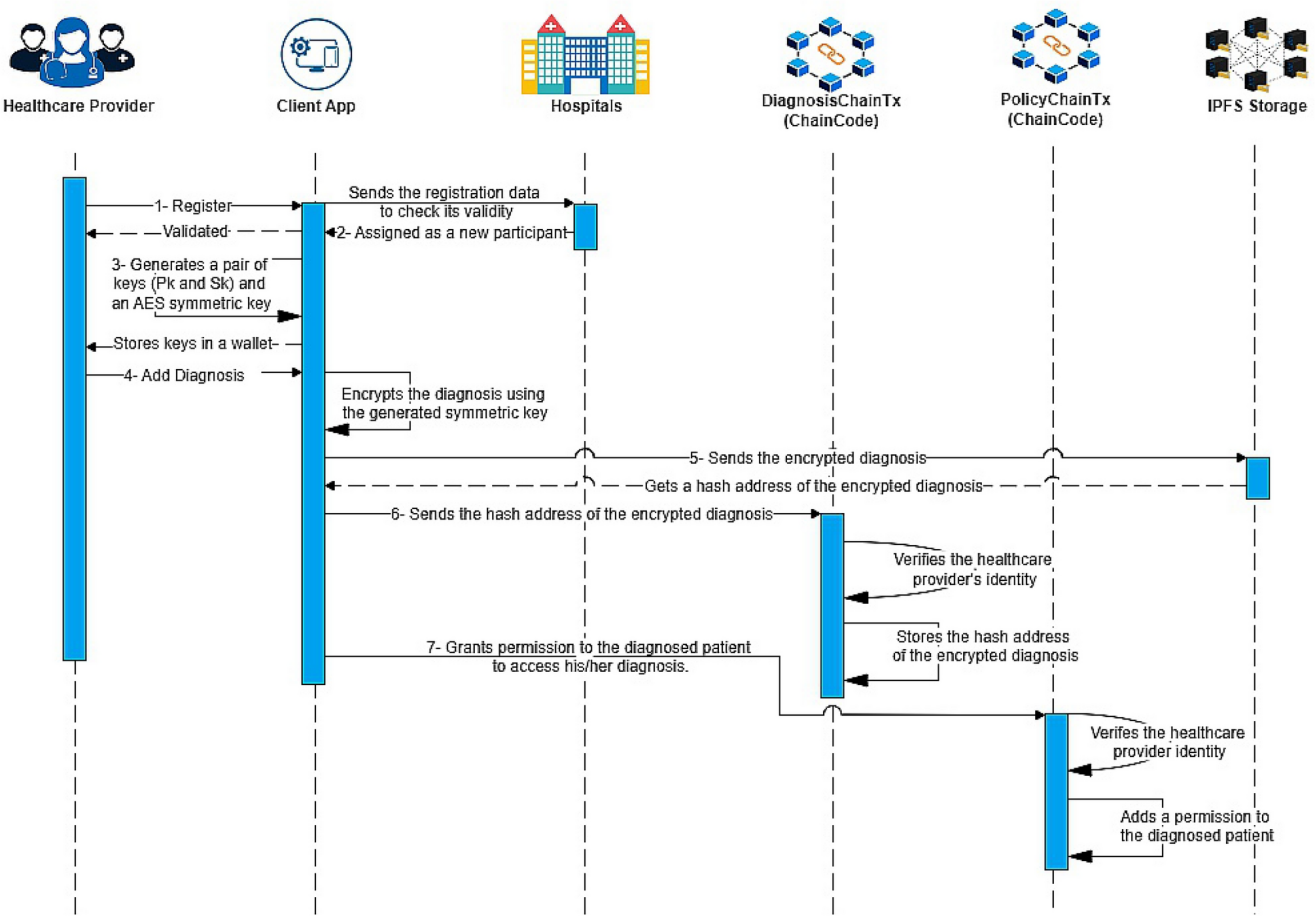
Healthcare providers scenario.

Healthcare providers can access and manage their patients’ EHRs, contributing diagnoses as authorized entities. Their interactions are governed by access control policies, as detailed in Algorithm 3. The healthcare provider scenario is illustrated in Fig. 3 and follows these steps:

1. The healthcare provider submits identity-related information to initiate a registration request.
2. Once the healthcare provider’s identity is validated, the healthcare provider is assigned as a new participant in the participating hospitals. At this point, a pair of keys (public key Pk and private key Sk) is generated for the healthcare provider and stored securely in the provider’s wallet.
3. The client app also generates an AES symmetric key to encrypt the patient’s diagnosis and stores it in the healthcare provider’s wallet.
4. The client app securely encrypts the added diagnosis by the healthcare provider, transmits it to an IPFS storage node, and retrieves the hash address associated with the patient’s diagnosis from IPFS.
5. The client app sends the hashed address of the patient’s diagnosis to the blockchain network.
6. The healthcare provider’s identity is verified using the DiagnosisChain chaincode within the permissioned blockchain. Once the verification is complete, the hashed address of the patient’s diagnosis is securely stored within the DiagnosisChain, which is a part of the overall blockchain network.
7. After the successful verification of the identity of the healthcare provider through the PolicyChain chaincode, the healthcare provider grants the diagnosed patient access to the diagnosis by incorporating a policy into PolicyChain.

**Fig. 4:**
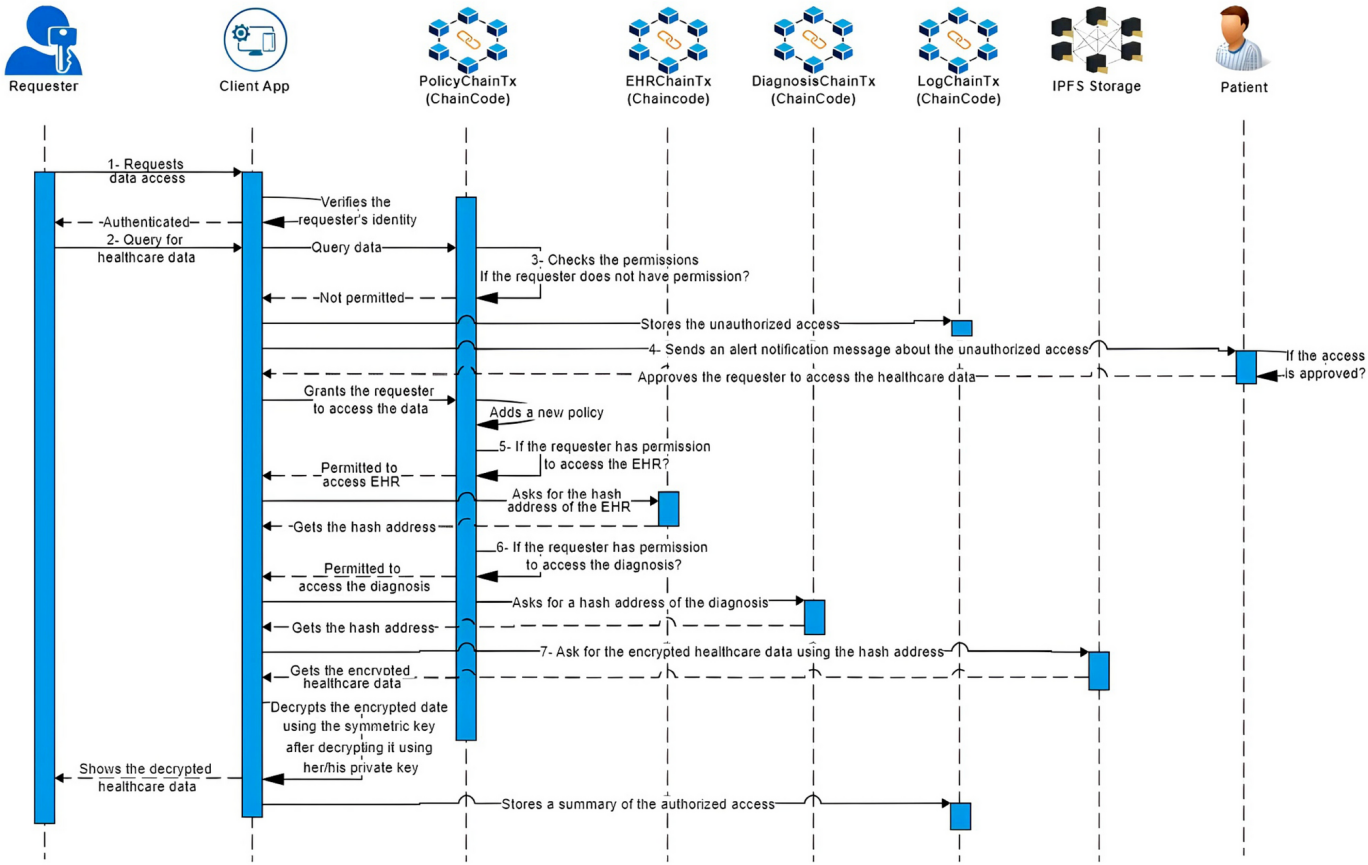
Healthcare data access scenario.

### Healthcare data access scenario

Healthcare data access refers to the ability of authorized individuals or entities to retrieve and view healthcare-related information, including EHRs and diagnoses, as detailed in Algorithm 4. Efficient data access enhances collaboration among healthcare providers, improves patient care coordination, and supports informed decision-making based on accurate and up-to-date information. The healthcare data access scenario is illustrated in Fig. 4 and follows these steps:

1. The client app receives a request from a requester, who can be either a patient or a healthcare provider, to access the relevant healthcare data.
2. Once the identity of the requester is authenticated, the client app verifies if the request for healthcare data is permitted by checking the PolicyChain, which acts as a permissioned blockchain. This ensures that access to the healthcare data is granted based on the defined permissions and policies within the blockchain network.
3. If the request for healthcare data is not permitted, such as when the requester’s policy is expired or invalid, the client app logs this unauthorized access to the LogChain. The LogChain acts as a permissioned blockchain, securely recording and storing details of unauthorized access attempts. Additionally, the client app sends an alert notification message to the relevant patients, informing them about the attempted access to their healthcare data without proper authorization.
4. When the patients receive the alert notification regarding the unauthorized access, if they decide to approve the access, the client app adds a new policy to the PolicyChain. This new policy includes an expiration time, validation, and specifies the type of EHR that the requester can access. By creating this new policy, the client app grants authorized access to the requester, enabling them to securely retrieve and access the patient’s healthcare data within the specified parameters.
5. If the request to access the patient’s EHR is permitted, the client app first retrieves the corresponding hashed address of the EHR from the EHRChain. Subsequently, the client app retrieves the encrypted EHR from the IPFS storage using the hashed address. To decrypt the EHR, the client app utilizes the requester’s private key to decrypt the enveloped symmetric key in the policy transaction and then obtains the symmetric key. By employing this key, the client app successfully decrypts the EHR, making it accessible for further use and analysis.
6. Similarly, if the request to access the patient’s diagnosis is granted, the client app acquires the corresponding hashed address of the diagnosis from the DiagnosisChain. Subsequently, the client app retrieves the encrypted diagnosis from the IPFS storage using the obtained hashed address. To decrypt the diagnosis, the client app utilizes the requester’s private key to decrypt the enveloped symmetric key in the policy transaction and then obtains the symmetric key. By employing this key, the client app successfully decrypts the diagnosis, making it accessible for further use.
7. The client app displays the requested healthcare data to the authorized requester. Additionally, the client app logs this authorized access to the LogChain, ensuring that a record of the access is securely stored within the permissioned blockchain for auditing and accountability purposes.

**Algorithm 4:**
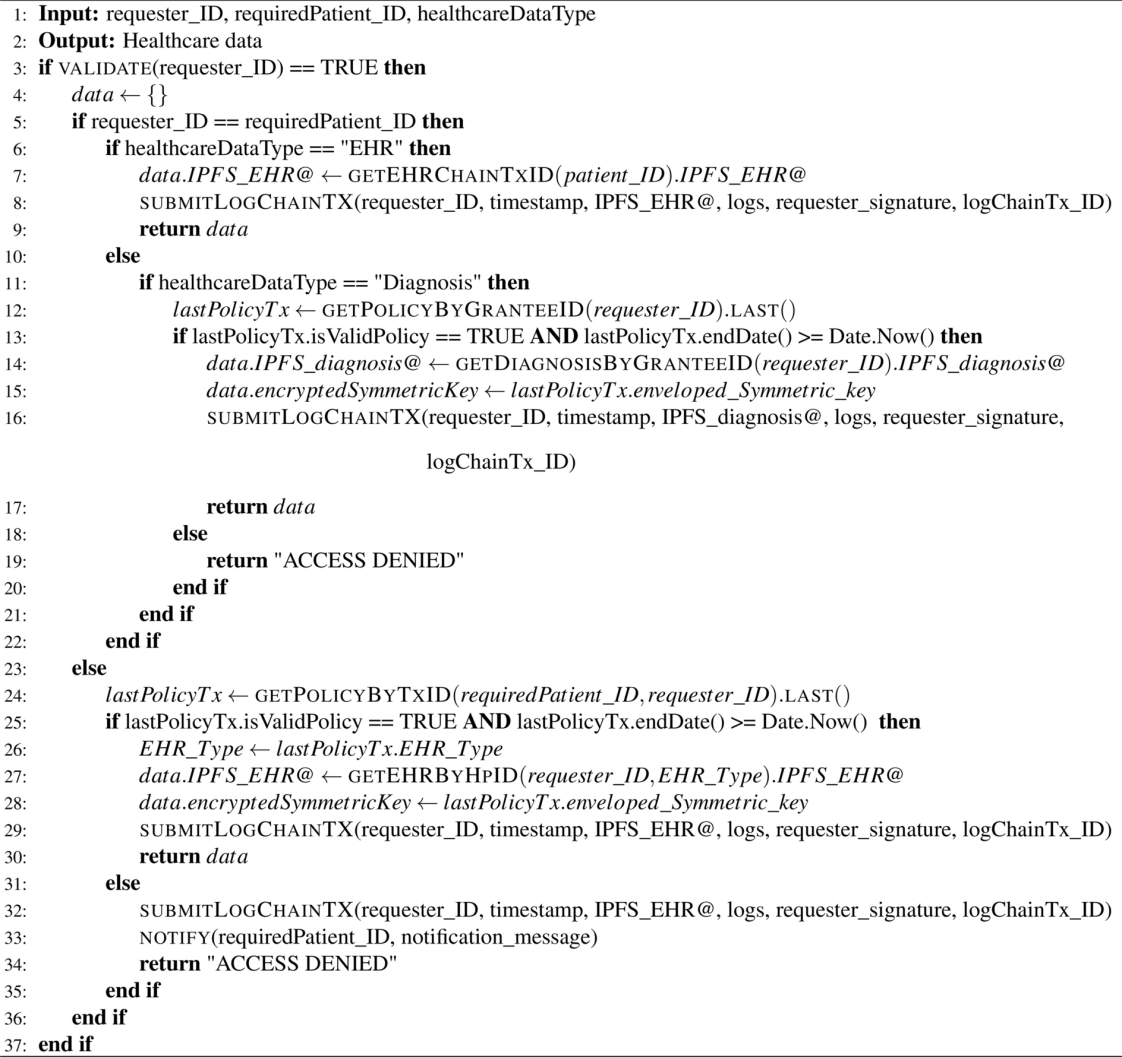
Chaincode of healthcare data access.

Having examined the implementation of ACHealthChain and its potential to enhance various healthcare scenarios, we now transition to a detailed evaluation of its security and privacy features. A robust security framework is essential for safeguarding sensitive medical data, ensuring patient confidentiality, and maintaining system integrity. In the following sections, we conduct a comprehensive analysis to assess how ACHealthChain mitigates potential threats, upholds data privacy, and withstands various security challenges inherent to blockchain-based healthcare systems.

## Security and privacy analysis

The Security and privacy Analysis section aims to evaluate the security and privacy aspects of the system.

### Security and threat model analysis

In this subsection, we analyze the security of ACHealthChain using the CIA triad (Confidentiality, Integrity, Availability) and accountability, augmented by formal threat models, including Dolev-Yao^39^. Additionally, we discuss possible attack scenarios and how ACHealthChain mitigates them.

- **Confidentiality:** Confidentiality ensures that only authorized requesters can access healthcare data. The ACHealthChain framework achieves this by employing a combination of Elliptic Curve Cryptography (ECC)^40^ and AES encryption^35^. Patients or healthcare providers use their AES symmetric key to encrypt data before storing it on IPFS storage. This key is then encrypted using the grantee’s ECC-generated public key and included in the granted policy transaction of the PolicyChain. To access encrypted data, authorized requesters retrieve it from IPFS using its hash address and decrypt it using the AES symmetric key associated with a valid policy in the PolicyChain. Prior to decryption, the requester’s private key decrypts the enveloped symmetric key. This process ensures that only authorized requesters with valid policies can decrypt the data, maintaining strong confidentiality measures to protect patient privacy.
- **Integrity:** The ACHealthChain framework ensures healthcare data integrity through blockchain immutability^10^. To ensure integrity, the framework compares the hash of the encrypted data stored on the ledger with the hash applied to the encrypted data retrieved from IPFS storage. If the hashes match, the data is considered intact and provided to the requester. Conversely, if the hashes do not match, indicating potential corruption, users are promptly notified of the discrepancy.
- **Availability:** The decentralized nature of the proposed framework eliminates the typical single point of failure concern, thereby improving overall availability when compared to centralized counterparts. Additionally, the utilization of IPFS decentralized storage for storing encrypted healthcare data ensures the guaranteed availability of the data.
- **Accountability:** Accountability ensures that authorized users’ actions, such as generating health records, can be audited and any malicious activities traced. In the ACHealthChain framework, both patients and healthcare providers are held accountable through their signatures stored in EHRChain and DiagnosisChain. Moreover, the framework enhances accountability through LogChain, which logs and monitors service execution based on smart contracts. Unauthorized access attempts are recorded in LogChain for auditing, aiding in the prevention of medical disputes and the identification of potential security breaches or suspicious activities. This robust logging mechanism ensures transparency in user actions and facilitates effective identification and mitigation of malicious activities within the system.

#### Threat model

In this subsection, we outline the potential security threats to the ACHealthChain framework. We analyze various attack vectors that could compromise the system’s integrity, confidentiality, and availability.

- **Unauthorized access attempts:** Malicious actors may attempt to gain unauthorized access to sensitive healthcare data stored within the ACHealthChain framework. This could involve exploiting vulnerabilities in the system or using stolen credentials to bypass authentication mechanisms, potentially resulting in unauthorized viewing, modification, or theft of patient information.
- **Denial of Service (DoS) attack:** Attackers may target the ACHealthChain network with a DoS attack, flooding it with a high volume of requests or malicious traffic, causing the system to become overwhelmed and unable to respond to legitimate requests. This could disrupt healthcare services, leading to delays in accessing patient records or processing transactions.
- **Eavesdropping attack:** In an eavesdropping attack, adversaries intercept and monitor communication between nodes or users within the ACHealthChain network, aiming to capture sensitive information such as patient diagnoses, treatment plans, or personal details. This breach of confidentiality could lead to privacy violations and expose patients’ confidential medical data to unauthorized parties.
- **Dolev-Yao model attack:** The Dolev-Yao adversary model assumes that an attacker can intercept, modify, and inject messages into the network. Such an attacker could attempt to manipulate communication between nodes within ACHealthChain, altering transactions or policy data to mislead participants into making incorrect access control decisions.
- **Sybil attack:** In a Sybil attack, an adversary generates multiple fake identities or nodes to gain an unfair influence over the consensus process. In ACHealthChain, an attacker could attempt to introduce a large number of malicious nodes to disrupt the validation of transactions or manipulate access control policies.

#### Attack scenarios

This subsection analyzes ACHealthChain’s resilience against five key security threats: (1) Unauthorized Access, bypassing access controls; (2) Denial-of-Service (DoS) Attacks, disrupting network availability; (3) Eavesdropping, intercepting sensitive data; (4) Dolev-Yao Network Threats, manipulating transactions via compromised communication channels; and (5) Sybil Attacks, forging multiple identities to gain undue influence.

**Scenario 1:** In this scenario, we consider the deployment of the ACHealthChain framework ($\mathscr {F}$) within a healthcare system where patients (*P*) and requesters (*R*) interact to manage and access healthcare data (*D*). Requesters *R* could represent healthcare providers or other entities attempting to access the data. We assume an unauthorized requester $R_N$ attempts to access healthcare data *D*.

##### Theorem 1

*The ACHealthChain framework effectively prevents Unauthorized Access Attempts.*

##### *Proof*

As depicted in Fig. 4 and Algorithm 4, $\mathscr {F}$ incorporates access controls, empowering patient *P* to manage and specify access to their data *D*. Upon receiving a request from requester *R*, peers verify the identity of *R* and query PolicyChain transactions (*C*) for associated policies, including a validation policy (*V*), then check for types of healthcare data (*T*), and expiration date ($D_e$). Let $S_{auth}(\mathscr {F}, P, R)$ represent the security property of authorization within the framework $\mathscr {F}$ between patients *P* and requesters *R*. Then,

$$\begin{aligned} S_{auth}(\mathscr {F}, P, R) = {\left\{ \begin{array}{ll} R_A, & \text {if } R = R_A \text { (Authorized)} \\ R_N, & \text {if } R = R_N \text { (Unauthorized)} \end{array}\right. } \end{aligned}$$

If the requester *R* is authorized $R_A$, the encrypted data (*E*) is retrieved from IPFS using a hash stored in either the EHRChain or DiagnosisChain. Subsequently, the encrypted data *E* is decrypted and provided to the authorized $R_A$ in adherence to the valid policy *C*. $\mathscr {F}$ detects and responds to unauthorized access $R_N$ through an alert notification message (*M*) to the patient *P*, preventing data breaches and ensuring compliance with the policy *C*.

$$\begin{aligned} P \leftarrow M(\mathscr {F}, P, R_N) \end{aligned}$$

$$\square$$

**Scenario 2:** In this scenario, we consider the deployment of the ACHealthChain framework ($\mathscr {F}$) within a healthcare system where multiple nodes (*N*) collaborate to manage and access healthcare data (*D*). We assume that an unauthorized requester ($R_N$) attempts to disrupt healthcare services by sending a high volume of requests.

##### Theorem 2

*The ACHealthChain framework mitigates DoS Attacks.*

##### *Proof*

To mitigate DoS attacks, $\mathscr {F}$ implements a distributed architecture and employs the PBFT consensus algorithm, which requires the agreement of at least $( \frac{{2 \times N}}{{3}})$ nodes to validate transactions. This distributed approach distributes the network load across *N* nodes, preventing any single point of failure and making it significantly more challenging for attackers to disrupt the network. Additionally, $\mathscr {F}$ is resilient against this attack because the identity of $R_N$ is checked, and the authenticity of received messages can be confirmed by the *N* nodes. This allows the *N* nodes to deny requests from $R_N$, ensuring the continuous availability of healthcare services. Let $S_{dos}(\mathscr {F}, N, R_N)$ represent the security property of mitigating DoS attacks within the ACHealthChain framework $\mathscr {F}$ with *N* nodes against unauthorized requester $R_N$. Then,

$$\begin{aligned} S_{dos}(\mathscr {F}, N, R_N) = R_A \text { (Authorized)} \end{aligned}$$

$$\square$$

**Scenario 3:** Consider the ACHealthChain framework ($\mathscr {F}$) deployed in a healthcare system where patients (*P*) and healthcare providers (*H*) interact to manage and access healthcare data (*D*). We assume an attacker ($\mathscr {A}$) tries to intercept the communication between nodes (*N*), patients *P*, or healthcare providers *H* within the $\mathscr {F}$ network, aiming to capture sensitive healthcare data (*D*).

##### Theorem 3

*The ACHealthChain framework ensures confidentiality and is resilient to Eavesdropping Attacks.*

##### *Proof*

$\mathscr {F}$ ensures confidentiality by combining ECC and AES encryption methods. Patients *P* or healthcare providers *H* use their AES symmetric key (*SK*) to encrypt data before storing it on IPFS storage. The *SK* is then encrypted using the grantee’s ECC-generated public key (*PK*) and included in the granted policy transaction of the PolicyChain. To access encrypted data (*E*), authorized requesters ($R_A$) retrieve it from IPFS using its hash address and decrypt it using the *SK* associated with a valid policy in the PolicyChain. Before decryption, the requester’s *PK* decrypts the enveloped *SK*. These encryption and security measures prevent adversaries, such as $\mathscr {A}$, from deciphering intercepted network traffic *D*, effectively disappointing eavesdropping attacks. Let $S_{conf}(\mathscr {F}, P, H, \mathscr {A}, N)$ represent the security property of ensuring confidentiality within the ACHealthChain framework $\mathscr {F}$ with patients *P*, healthcare providers *H*, attacker $\mathscr {A}$, and *N* nodes. Then,

$$\begin{aligned} S_{conf}(\mathscr {F}, P, H, \mathscr {A}, N) = R_A \text { (Authorized)} \end{aligned}$$

$$\square$$

**Scenario 4:** Consider the ACHealthChain framework ($\mathscr {F}$), where an adversary ($\mathscr {A}_{DY}$) operates under the Dolev-Yao model^39^. This adversary can intercept messages between patients (*P*) and healthcare providers (*H*), inject fabricated transactions into PolicyChain (*C*), and replay old access requests to manipulate system behavior.

##### Theorem 4

$\mathscr {F}$ *resists Dolev-Yao adversaries through cryptographic message authentication.*

##### *Proof*

Each network message in $\mathscr {F}$ is structured as:

$$\begin{aligned} m = \langle E_{PK}(SK), Sig_{K^-}(H(E)), T_s \rangle \end{aligned}$$

where $T_s$ is a fresh timestamp, and $Sig_{K^-}$ represents a digital signature using the sender’s private key. Let $S_{dy}(\mathscr {F}, \mathscr {A}_{DY})$ denote the system’s resilience against Dolev-Yao attacks:

$$\begin{aligned} S_{dy}(\mathscr {F}, \mathscr {A}_{DY}) = {\left\{ \begin{array}{ll} 0 & \text {if } \mathscr {A}_{DY} \vdash E_{PK}(SK) \text { or } Sig_{K^-}(H(E)) \\ 1 & \text {otherwise} \end{array}\right. } \end{aligned}$$

The framework maintains $S_{dy} = 1$ due to the following security measures: (i) timestamps $T_s$ prevent replay attacks by ensuring that old messages are not reused; (ii) ECC-based digital signatures validate message authenticity, preventing unauthorized message injections; and (iii) encrypted symmetric keys (*SK*) remain secure under Dolev-Yao assumptions, ensuring confidentiality even if adversaries intercept network communications. Thus, $\mathscr {F}$ successfully mitigates Dolev-Yao threats.

$$\square$$

**Scenario 5:** In this scenario, an adversary ($\mathscr {A}_S$) creates multiple fake identities $\{R_{S_1},...,R_{S_k}\}$ to manipulate the ACHealthChain framework ($\mathscr {F}$). The attacker may attempt to influence the PBFT consensus protocol by controlling more than $\frac{N}{3}$ nodes or bypass access control mechanisms using fraudulent credentials.

##### Theorem 5

$\mathscr {F}$ *prevents Sybil attacks through MSP-based identity management.*

##### *Proof*

To counter Sybil attacks, the MSP component of $\mathscr {F}$ enforces strict identity verification. Each requester *R* is issued a unique certificate by a trusted CA, ensuring that no entity can generate multiple identities fraudulently:

$$\begin{aligned} \forall R \in \mathscr {R}, \exists ! \ cert_R \text { where } Verify_{CA}(cert_R) = 1 \end{aligned}$$

Let $S_{sybil}(\mathscr {F}, \mathscr {A}_S, k)$ represent the framework’s resistance to Sybil attacks:

$$\begin{aligned} S_{sybil}(\mathscr {F}, \mathscr {A}_S, k) = \frac{|\{cert_{S_i} | Verify_{CA}(cert_{S_i}) = 1\}|}{k} \end{aligned}$$

The framework achieves $S_{sybil} = 0$ by implementing: (i) the CA that ensures a one-to-one mapping between real-world entities and digital identities; (ii) PBFT consensus, which requires at least $\frac{2N}{3}$ legitimate nodes to approve transactions, making Sybil attacks impractical; and (iii) PolicyChain validation, where access control policies enforce strict authentication based on MSP records. These mechanisms prevent adversaries from successfully executing Sybil attacks within $\mathscr {F}$. $\square$

### Privacy analysis

In this subsection, we explore the privacy considerations within our proposed framework.

- **Access control:** The ACHealthChain framework incorporates granular access controls, empowering patients to manage and specify access to their healthcare data. Policies and permissions are enforced to prevent unauthorized access, with access authorization governed by policies defined in the PolicyChain. Peers, acting as endorsers, play a crucial role in regulating requester access to patient healthcare data and ensuring compliance with PolicyChain transactions. Upon receiving a request transaction, including patient ID, EHR type, and data duration, endorsers verify its validity and query the PolicyChain for associated policies. If authorized, the client app retrieves encrypted healthcare data from IPFS using a hash stored in either the EHRChain or DiagnosisChain. Subsequently, the data is decrypted and provided to the requester in adherence to valid PolicyChain policies.
- **Revocability:** Patients have the capability to revoke healthcare providers’ access to their healthcare data through the PolicyChain. This process involves defining a set of grantees with validation, an expiration date, and an EHR type for accessing their data. If the requesters possess valid access permissions, the chaincode allows the retrieval of encrypted data from IPFS storage using the hash address from either the EHRChain or DiagnosisChain. Subsequently, the data is decrypted using the AES symmetric key, which is accessed with the requester’s private key. In case a patient revokes access for a healthcare provider, a new policy transaction is added with a false value in the validation parameter. The chaincode of the PolicyChain verifies this parameter in the last policy transaction related to the patient and healthcare provider. If access is revoked via the PolicyChain with a false validation parameter, the healthcare provider can no longer access the data unless granted new permission by the patient. Conversely, if the validation parameter is true, the chaincode checks for the expiration date and EHR type. This mechanism ensures that patients can effectively manage access permissions, thereby maintaining control over their healthcare data and enhancing overall data security.
- **Alert notifications:** The ACHealthChain framework integrates an alert notification system designed to promptly notify patients of any unauthorized access attempts to their healthcare data. This proactive feature empowers patients by providing timely information, enabling them to make informed decisions regarding access to their data. Upon receiving these notifications, patients have the option to approve or decline the access attempt, thus retaining control over their privacy and ensuring the security of their healthcare information. This functionality allows patients to take necessary actions aligned with their preferences and concerns, facilitating active management and protection of their data within the ACHealthChain framework.

After thoroughly analyzing the security and privacy aspects of the ACHealthChain framework in different scenarios and providing evidence of its effectiveness, we now focus on the evaluation and experimental results. In the next section, we present empirical evidence from various experiments conducted to assess the system’s scalability, efficiency, and robustness using Hyperledger Fabric Blockchain.

## Evaluation and experimental results

In order to evaluate the performance of our ACHealthChain framework, we deployed the business network scenario on Hyperledger Fabric v2.4.7, an open-source, modular blockchain platform for enterprise applications. Privacy was ensured using Fabric channels^13^, where participants share a common ledger and chaincodes for specific business purposes. ACHealthChain integrates IPFS for decentralized storage and scalability while employing the PBFT protocol with Raft ordering service^37^ for consensus. Raft, a crash fault-tolerant ordering service in Fabric, outperforms Kafka and Solo in scalability and performance by enabling multiple nodes to jointly manage transaction ordering. Its advantages-including fault tolerance, leader election, and simplified configuration-make Raft ideal for production environments, streamlining the deployment and management of ordering nodes.

**Table 2:** Comparison between ACHealthChain and related work from privacy, security, and attack resistance perspectives.

Reference	Confidentiality	Integrity	Availability	Decentralized	Accountability	Revocability	Scalability	UAA	DoS	EA	DY	SA
MedRec^22^	$\checkmark$	✗	✗	$\checkmark$	$\checkmark$	✗	✗	$\checkmark$	✗	✗	✗	✗
MeDShare^23^	$\checkmark$	$\checkmark$	✗	✗	✗	$\checkmark$	$\checkmark$	$\checkmark$	$\checkmark$	$\checkmark$	✗	✗
$\hbox {MediChain}^{TM}$ ^24^	$\checkmark$	$\checkmark$	✗	✗	✗	✗	$\checkmark$	$\checkmark$	✗	✗	✗	✗
Healthchain^25^	$\checkmark$	$\checkmark$	$\checkmark$	$\checkmark$	$\checkmark$	$\checkmark$	✗	$\checkmark$	$\checkmark$	$\checkmark$	✗	$\checkmark$
SPChain^26^	$\checkmark$	$\checkmark$	✗	$\checkmark$	✗	✗	✗	$\checkmark$	$\checkmark$	$\checkmark$	✗	$\checkmark$
BCHealth^27^	$\checkmark$	$\checkmark$	✗	✗	✗	$\checkmark$	✗	$\checkmark$	$\checkmark$	$\checkmark$	✗	✗
Xiang and Zhao^28^	$\checkmark$	$\checkmark$	✗	✗	✗	✗	✗	$\checkmark$	✗	$\checkmark$	✗	✗
Diaz et al.^29^	$\checkmark$	$\checkmark$	$\checkmark$	✗	✗	✗	✗	$\checkmark$	$\checkmark$	✗	✗	$\checkmark$
Lax et al.^30^	$\checkmark$	$\checkmark$	$\checkmark$	$\checkmark$	✗	✗	✗	$\checkmark$	✗	$\checkmark$	$\checkmark$	✗
ACHealthChain	$\checkmark$	$\checkmark$	$\checkmark$	$\checkmark$	$\checkmark$	$\checkmark$	$\checkmark$	$\checkmark$	$\checkmark$	$\checkmark$	$\checkmark$	$\checkmark$

Attack resistance columns: UAA=Unauthorized Access Attempts, DoS=Denial of Service Attack, EA=Eavesdropping Attack, DY=Dolev-Yao Model Attack, SA=Sybil Attack

### Functional analysis

In this subsection, we conduct a comprehensive comparison between the ACHealthChain framework and other related works^22–30^ based on various critical metrics: confidentiality, integrity, availability, decentralization, accountability, revocability, scalability, and attack resistance, as illustrated in Table 2. We use the symbols ($\checkmark$) to denote compliance with a metric and (✗) to indicate non-compliance. Notably, most of the reviewed studies satisfy the metrics of confidentiality and integrity, while scalability remains a challenge for the majority. Additionally, several frameworks, such as MeDShare^23^ and Healthchain^25^, implement access revocation mechanisms, whereas decentralized architectures are present in only a subset of the studied works. This comparison builds upon the insights provided in section “Related work” and Table 1, where these studies employ diverse consensus protocols, such as PoW and PBFT, while implementing various privacy techniques, including smart contracts, proxy re-encryption, and ABE.

Regarding attack resistance, we assess each framework’s ability to counter unauthorized access attempts (UAA), denial-of-service attacks (DoS), eavesdropping attacks (EA), Dolev-Yao model attacks (DY), and Sybil attacks (SA). As shown in Table 2, ACHealthChain outperforms other frameworks by providing resistance to all five attack types. While some existing solutions offer partial resistance-such as Healthchain^25^, SPChain^26^, and Diaz et al.^29^, which mitigate Sybil attacks, and Lax et al.^30^, which only addresses EA attacks-none comprehensively protect against all categories of attacks. As demonstrated in section “Security and privacy analysis”, our framework satisfies all security and functional metrics, ensuring a robust and scalable solution. Therefore, ACHealthChain exhibits superior security and functionality compared to existing frameworks.

### Experimental setup

The experiments were conducted in a controlled test environment, as summarized in Table 3. The ACHealthChain framework was deployed on a single-machine setup running Ubuntu Linux 22.04 LTS, with a Hyperledger Fabric 2.4.7 network consisting of five organizations, each with one peer and three clients. The Raft-based ordering service was utilized for consensus, and CouchDB was employed as the on-chain state database. Off-chain data storage was managed using IPFS, ensuring efficient and decentralized storage of electronic health records. To evaluate ACHealthChain’s performance, we leveraged Hyperledger Caliper, a benchmarking tool designed for measuring key performance metrics such as scalability, throughput, latency, and resource utilization^41^. Caliper, part of the Hyperledger project, provides an extensible and standardized framework for assessing blockchain-based applications across different platforms, including Hyperledger Fabric and Ethereum. Within our implementation, Caliper was integrated via the Fabric Client SDK in Java, enabling direct interaction with the blockchain network for executing and analyzing transactions.

**Fig. 5:**
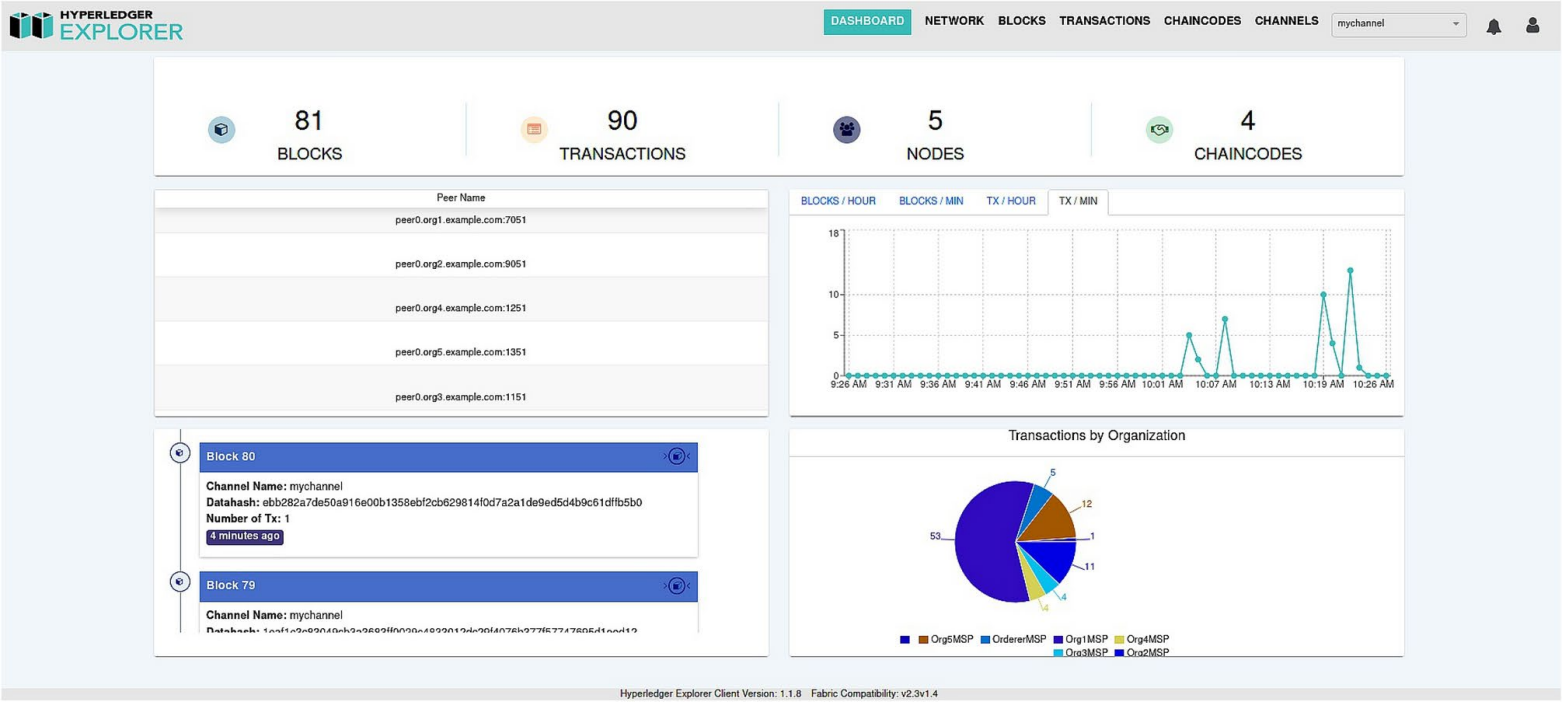
An overview of the proposed framework using Hyperledger Explorer^42^.

For network monitoring and visualization, we employed Hyperledger Explorer^42^, a web-based tool that allows real-time inspection of blocks, transactions, chaincodes, and network activity. An overview of the proposed framework’s deployment within Hyperledger Explorer is illustrated in Fig. 5, showcasing its operational structure and transaction flow.

**Table 3:** Experimental test environment .

Component	Configuration
Distribution	Single machine
Machine operating system	Ubuntu Linux 22.04 LTS
CPU	Intel Core i7, 2.8 GHz
Storage	16 GB memory, 256 GB SSD
Platform	Hyperledger Fabric 2.4.7
Size	5 Orgs with 1 peer and 3 clients
Orderer Service	Raft-based ordering service
On-chain	CouchDB
Off-chain	IPFS distributed storage

The performance evaluation encompassed a diverse set of healthcare providers, patients, and healthcare data records to ensure representative results. Synthetic access control requests were generated to simulate real-world scenarios. The experimental configuration consisted of the following components:

1. The IPFS off-chain distributed storage system was utilized to store encrypted healthcare data.
2. There are five organizations involved in the system: Org1, Org2, Org3, Org4, and Org5. Each organization represents a hospital and is equipped with the following components:
  One peer (Peer0): This peer is responsible for maintaining a copy of the blockchain ledger and participating in the consensus process within its organization.
  One Certificate Authority: This authority is responsible for issuing and managing digital certificates for secure communication and identity verification within the network.
  An on-chain database (CouchDB): This database is used to store the healthcare data records and provide efficient querying capabilities.
  Clients: Each organization has at least three types of clients:
    **Hospital admin:** This client represents the administrative staff of the hospital and has access to system management and configuration functionalities.
    **Healthcare provider:** This client represents the healthcare professionals within the hospital and can interact with the system to access and update patient data.
    **Patient:** This client represents the individuals receiving healthcare services and can access their own medical records and grant permission for data sharing.
3. One orderer service: This component is responsible for establishing consensus among network participants and ordering transactions into a consistent ledger. It acts as the central authority for the ordering and sequencing of transactions in a blockchain network. The orderer service ensures that all transactions are agreed upon by the network’s endorsing peers and orders them into a sequence known as a block. This block is then distributed to the peers for validation, execution, and updating their local copy of the ledger.
4. One channel hosts four permissioned sub-blockchains: EHRChain, DiagnosisChain, PolicyChain, and LogChain, each with its chain of blocks and real state (CouchDB).

**Table 4:** Network configuration for phase 1 evaluation.

Parameters	Configuration
Number of channels	01
Number of Orgs	05
Number of peers	05
Number of clients	06
Number of orderer services	01
Number of transactions (TXs)	50
Send Rate (TPS)	50 tps
Number of rounds	30

#### Experimental phases

To conduct performance experiments using Hyperledger Caliper and our custom Fabric Client SDK interface, we define the experimental phases as follows:

1. **Phase 1: Performance metrics of blockchains:** The primary objective of this initial phase is to evaluate various performance indicators of our network, particularly average Throughput (transactions per second), average Latency (response time), and Resource Consumption (CPU and memory usage) for each blockchain in our framework (EHRChain, DiagnosisChain, PolicyChain, and LogChain). This evaluation is based on the network configuration detailed in Table 4.
2. **Phase 2: Scalability analysis vs number of nodes:** In the second phase of the experiments, we focused on investigating the scalability and performance of the proposed ACHealthChain framework. Specifically, we examined how the system performs when the number of peer nodes is increased while keeping the other parameters consistent with those in phase 1. The number of peers varied from 2 to 26 in our study with a send rate of 200 TPS. By varying the number of peer nodes, we aimed to assess the impact of scalability on the system’s performance. This allowed us to understand how the solution performs under different levels of network growth and resource utilization.
3. **Phase 3: Transaction scalability analysis:** In this phase, we extended our study to evaluate the scalability of our framework by conducting a comparative analysis with the scheme proposed by Ucbas et al.^43^. We examined a transaction range from 100 to 5000 inputs while maintaining a fixed send rate of 50 TPS. The primary goal of this phase was to assess how increasing the number of input transactions affects transaction throughput and latency. By systematically adjusting the transaction volume, we aimed to gain a deeper understanding of the framework’s capacity to handle growing demands and to identify its performance limits in terms of throughput and latency. This analysis provides valuable insights into the scalability and efficiency of ACHealthChain, especially in comparison to established solutions.
4. **Phase 4: Scalability analysis vs users count:** In the fourth phase of our experiments, we focus on analyzing the scalability of the ACHealthChain framework by increasing the number of concurrent transactions from 10 users to 50 users. The objective is to assess the system’s performance as the number of concurrent transactions increases, considering various user counts.

**Table 5:** Configuration settings for phase 5 experiments.

Parameters	Configuration
Number of channels	01
Number of input TXs	1000
Tx send rate (TPS)	25, 50, 100, 150, 200
Number of peers	05
Number of orderer service nodes	1
Number of clients	06
Number of rounds	10

5. **Phase 5: Performance analysis vs transaction send rates:** In this experimental phase, we aimed to compare the performance of ACHealthChain with the scheme proposed by Díaz et al.^29^ to analyze the impact of varying transaction send rates on throughput and latency. By adjusting the send rate from 25 to 200 TPS, as outlined in Table 5, we sought to evaluate the scalability and responsiveness of ACHealthChain in processing large transaction volumes. Ten rounds of experiments were conducted at each send rate, and the average results were calculated to ensure reliability.

**Fig. 6:**
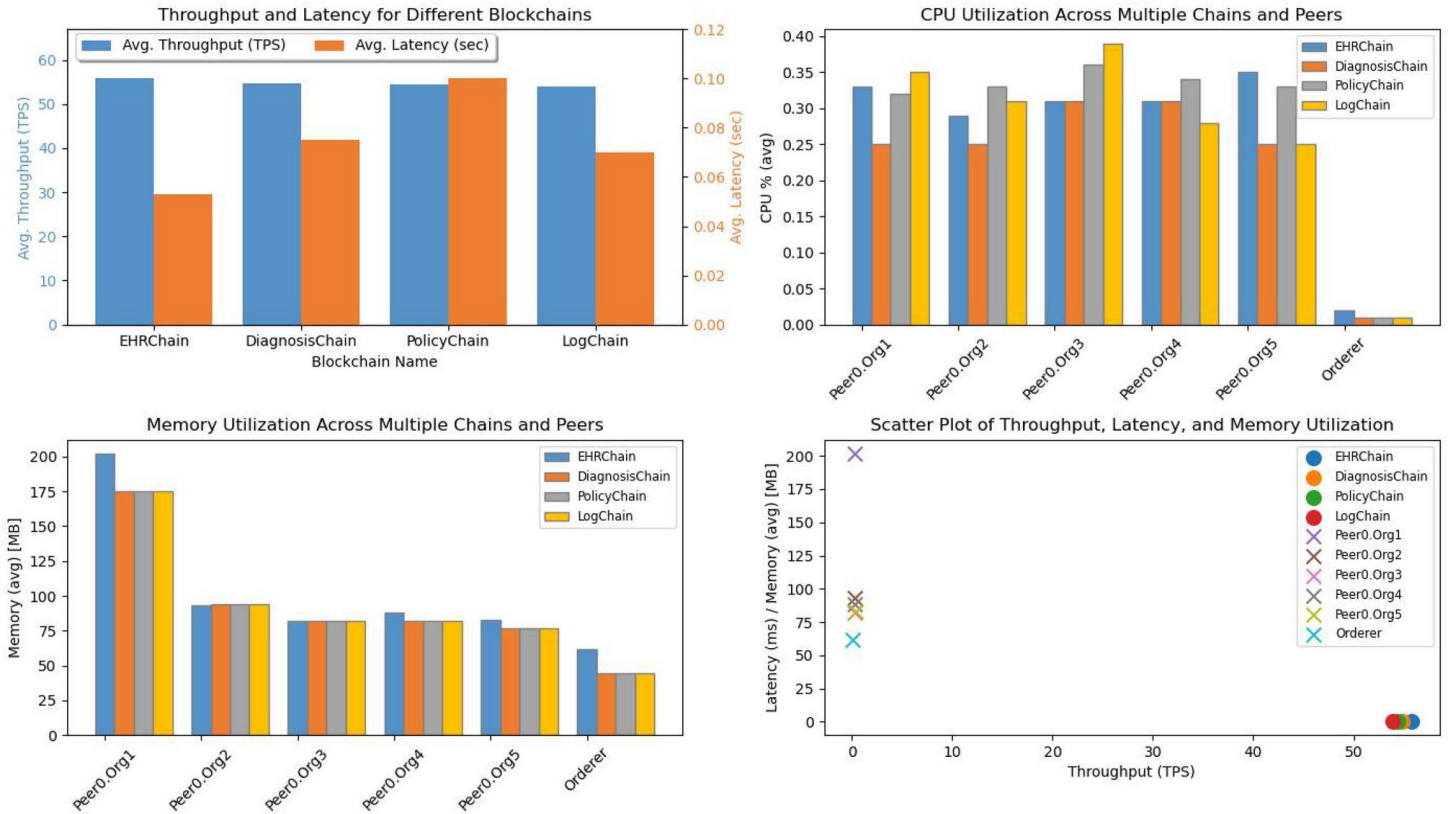
Performance evaluation of four blockchain platforms within the ACHealthChain framework.

### Results and discussion

The implementation and evaluation of the framework yielded the following results:

1. **First phase:** The initial stage of our experiment provided the following insights, as illustrated in Fig. 6. In subplot (1) of Fig. 6, we noted minor variations in average throughput (ranging from 53.9 to 55.8 TPS) and latency (ranging from 0.053 to 0.1 s) across different blockchains. Notably, EHRChain exhibited the highest throughput and lowest latency, while PolicyChain demonstrated the highest latency, likely due to its involvement in transactions related to EHRs or diagnoses. Subplots (2) and (3) in Fig. 6 depict varying CPU and memory utilization across chains and peers. We observed that resource consumption patterns aligned with the observed throughput and latency metrics. Notably, memory utilization was highest in Org1, serving as the initiator node for transactions in this experiment. Subplot (4) visualizes the relationships between throughput, latency, and memory utilization. These findings underscore the benefits of ACHealthChain’s approach, which leverages a single channel with four blockchains to facilitate communication and data exchange. By consolidating blockchains within a single channel, ACHealthChain effectively mitigates transaction latency issues and enhances overall throughput. Furthermore, these results emphasize the importance of tailoring blockchain solutions to meet specific healthcare application requirements. ACHealthChain’s resource-efficient nature positions it as a promising solution for practical deployment in healthcare data-sharing scenarios.

**Fig. 7:**
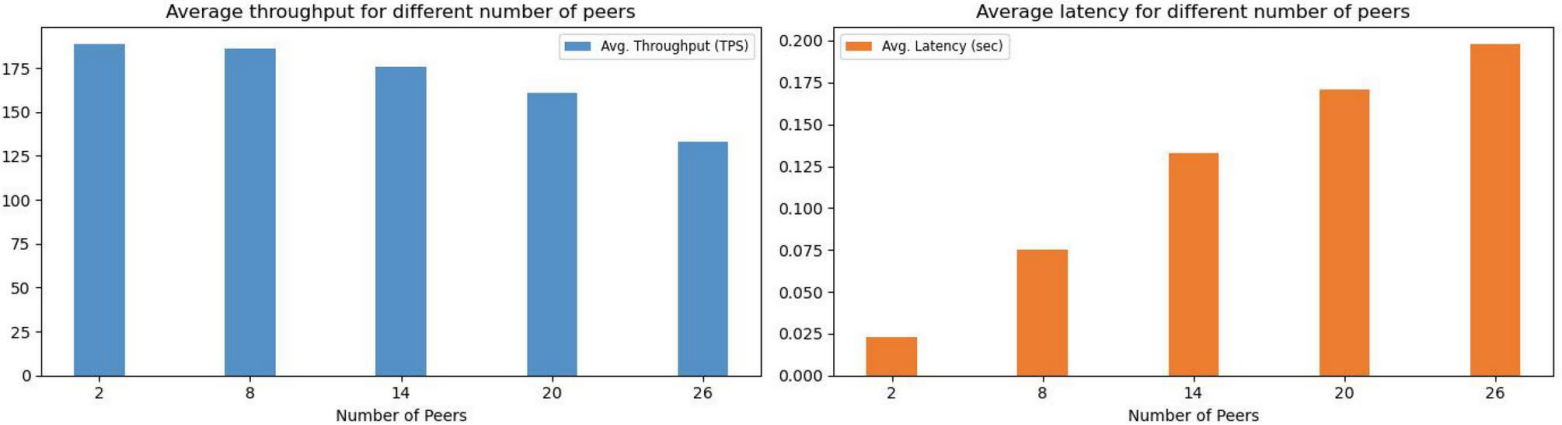
System performance and scalability analysis with varying numbers of peers.

**Fig. 8:**
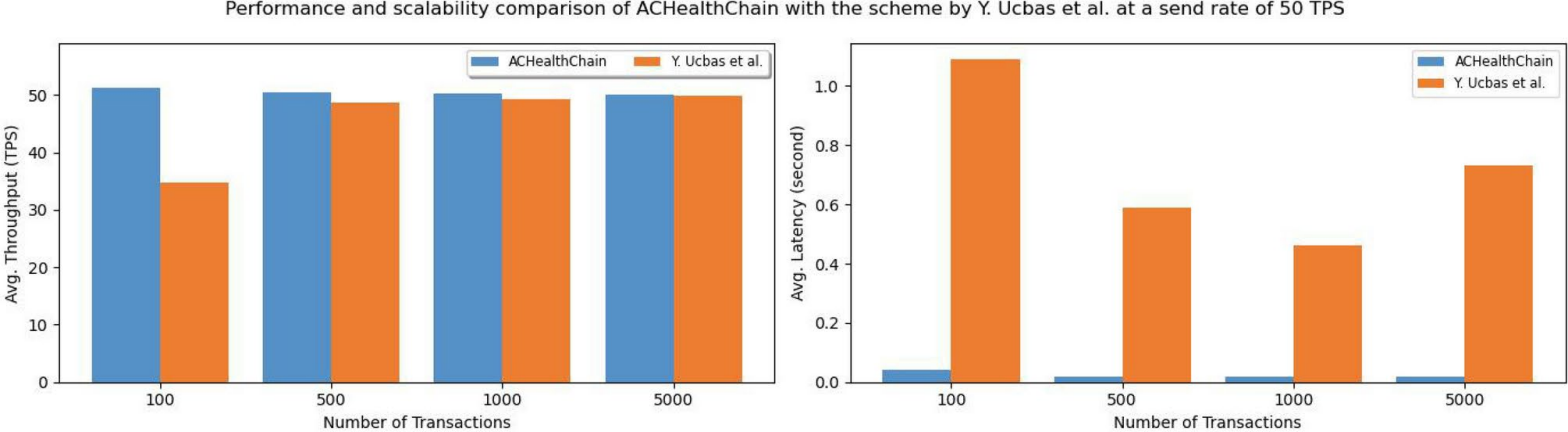
Performance and scalability comparison of ACHealthChain with the scheme proposed by Y. Ucbas et al.^43^ at a transaction send rate of 50 TPS.

2. **Second phase:** In this phase of the experiments, we observed a decrease in throughput and an increase in latency as the blockchain network scaled up, as depicted in Fig. 7. With the number of peer nodes increasing from 2 to 26, a clear downward trend in throughput emerged, declining from 188.64 TPS with 2 peer nodes to 132.73 TPS with 26 peer nodes. Concurrently, average latency increased from 0.023 sec to 0.198 sec across the same node range. This outcome stemmed from the heightened number of messages exchanged between nodes and the subsequent waiting time for endorsing and packaging those messages into blocks. As the network size expanded, communication overhead intensified, resulting in higher latency and lower throughput, particularly evident when deployed on a virtual machine with a single host. Nevertheless, it’s noteworthy that the decrease in throughput was relatively minor, showcasing the flexible scalability of the ACHealthChain framework. These findings underscore the framework’s adeptness in accommodating an expanding network without significantly compromising its performance, emphasizing its flexible scalability and effectiveness in meeting the demands of a growing blockchain network.

**Fig. 9:**
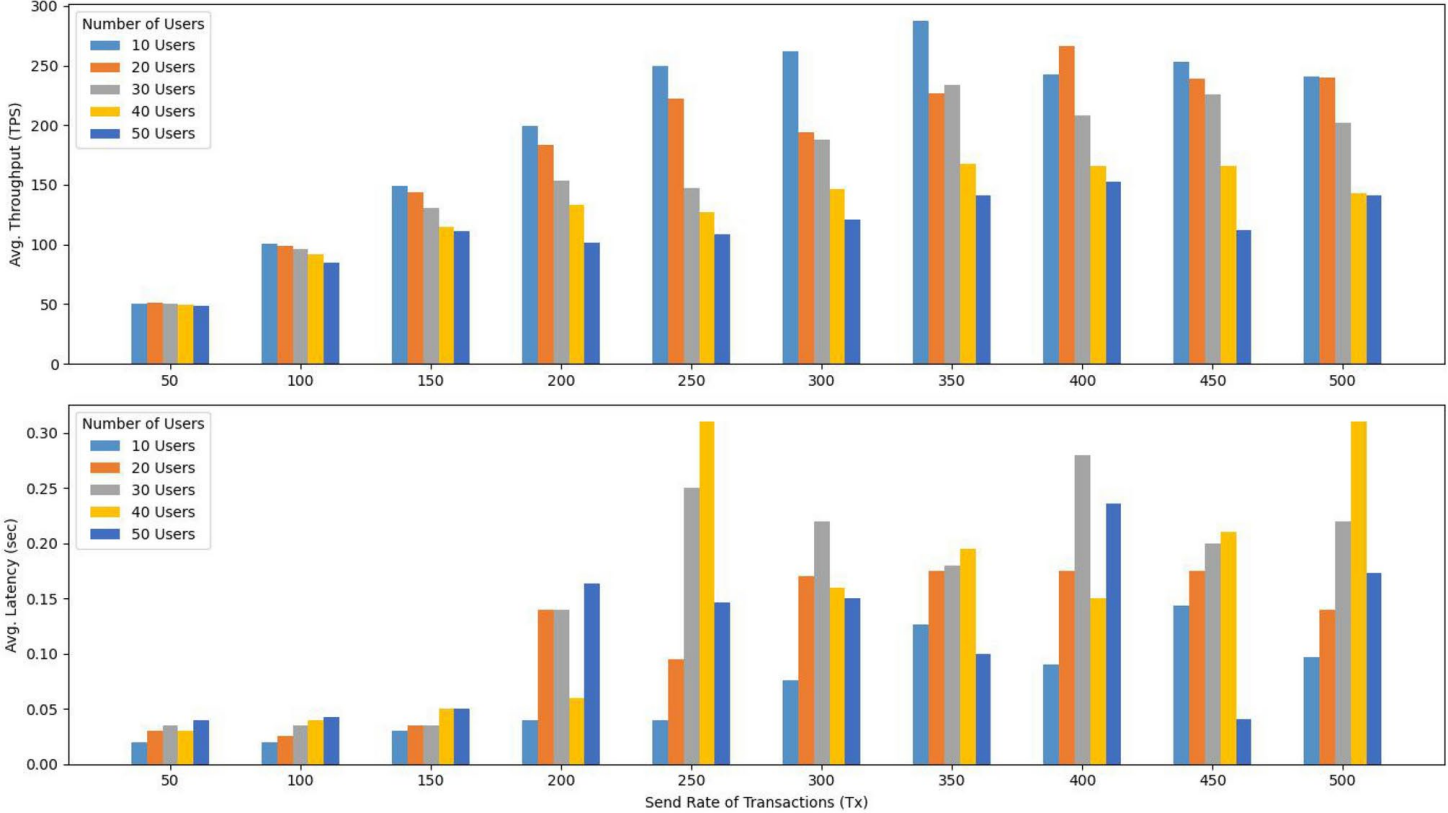
Scalability evaluation of ACHealthChain based on the number of users.

3. **Third phase:** In the third phase of our experiments, we performed a transaction scalability analysis by comparing ACHealthChain with the scheme proposed by Ucbas et al.^43^ across a range of 100 to 5000 input transactions, maintaining a fixed send rate of 50 TPS. The objective was to evaluate how each system performs in terms of throughput and latency as transaction volume scales up. As shown in Fig. 8, ACHealthChain consistently achieved higher throughput across all transaction ranges compared to the scheme by Ucbas et al. For instance, with 100 transactions, ACHealthChain reached approximately 51.3 TPS, surpassing Ucbas et al.’s 34.75 TPS. Even as the transaction volume increased to 5000, ACHealthChain maintained an optimal throughput close to 50 TPS, demonstrating minimal degradation. In contrast, Ucbas et al.’s scheme exhibited a throughput that plateaued near 49.9 TPS, showing limited scalability at higher transaction loads.

Similarly, latency measurements further underscored ACHealthChain’s superior performance. The latency for ACHealthChain remained exceptionally low, stabilizing at around 0.02 seconds, even as the transaction count scaled up to 5000. Conversely, Ucbas et al.’s scheme showed significantly higher latencies, starting at 1.09 seconds for 100 transactions and only reducing to 0.73 seconds at 5000 transactions. This substantial difference in latency underscores ACHealthChain’s ability to handle large transaction volumes with minimal delay, which is crucial for real-time healthcare applications. These results demonstrate that ACHealthChain not only supports high throughput but also achieves substantially lower latency than Ucbas et al.’s framework, affirming its scalability and efficiency in high-demand settings. The consistent performance across a wide range of transaction loads highlights ACHealthChain’s capacity to maintain robust, fast, and reliable service as transaction demands increase, making it highly suitable for scalable, blockchain-based healthcare systems.

**Fig. 10:**
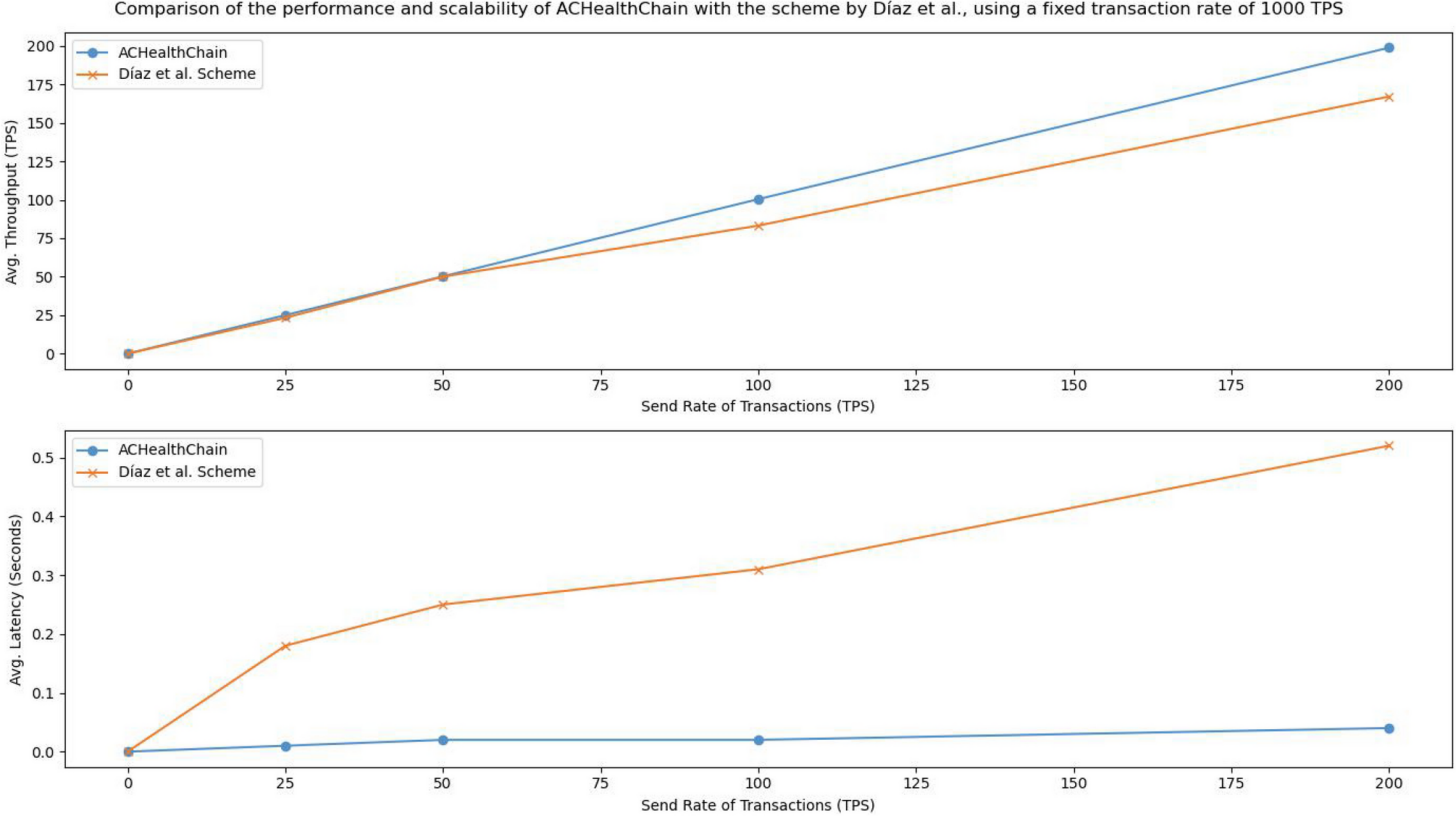
Comparison of ACHealthChain’s performance and scalability with the scheme proposed by Díaz et al.^29^, using a fixed transaction rate of 1000 TPS.

4. **Fourth phase:** In the fourth phase of our experiments, we focused on analyzing the scalability of the ACHealthChain framework concerning the number of users. The results, as depicted in Fig. 9, clearly demonstrate the average throughput and latency across varying user counts (10 to 50) and increasing transaction send rates. We observed a slight decrease in throughput and a slight increase in latency as the number of users rises, indicating that increasing users correspond to increasing concurrent transactions. Our experiment revealed that average throughput increases with the rising send rates until 350 TPS, after which it decreases slightly when deployed on a single machine with various dockers. However, deploying it on different nodes yields better results. These findings underscore the scalability and performance of ACHealthChain in handling an increased number of users, essential capabilities for applications requiring fast and reliable access to healthcare data.
5. **Fifth phase:** The fifth and final phase of our experiments, as shown in Fig. 10, reveals significant performance advantages of ACHealthChain over the Díaz et al. scheme^29^ when analyzing throughput and latency at different transaction send rates.

The top graph in Fig. 10 illustrates the throughput results, where ACHealthChain consistently outperforms Díaz et al.’s scheme across all tested send rates. For instance, at the highest send rate of 200 TPS, ACHealthChain achieves an average throughput close to 200 TPS, while Díaz et al.’s scheme only reaches approximately 167 TPS. This consistent advantage indicates that ACHealthChain can handle higher transaction volumes with greater efficiency, underscoring its superior scalability. In terms of latency, depicted in the bottom graph, ACHealthChain demonstrates significantly lower latency compared to Díaz et al.’s scheme across all send rates. While the latency in Díaz et al.’s framework sharply increases with higher send rates (reaching around 0.52 seconds at 200 TPS), ACHealthChain maintains a remarkably low and stable latency, peaking at just 0.04 seconds even at the maximum send rate. This low latency highlights ACHealthChain’s efficiency in transaction processing, making it more responsive under high transaction loads.

These findings underscore ACHealthChain’s ability to efficiently manage high transaction volumes with minimal latency, making it highly suitable for healthcare data management applications that demand both high throughput and low latency. ACHealthChain’s design, which utilizes a single channel with four permissioned subchains, effectively addresses the challenges associated with traditional multi-channel approaches. For real-world healthcare data-sharing scenarios, we recommend deploying ACHealthChain on high-performance, distributed servers to further enhance its scalability and robustness. This would optimize ACHealthChain for large-scale healthcare applications, where rapid, reliable data access is critical.

## Conclusion

This paper presents ACHealthChain, a blockchain-based framework leveraging Hyperledger Fabric for secure and efficient healthcare data access control. ACHealthChain introduces four separate subchains-EHRChain, DiagnosisChain, PolicyChain, and LogChain-enhancing security, privacy, and auditability while eliminating reliance on centralized authorities. Experimental results demonstrate that ACHealthChain increases throughput by 19.7% and reduces latency by 87% compared to existing frameworks. At a transaction rate of 200 TPS, it achieves a peak throughput of 200 TPS, surpassing the Díaz et al. scheme, which reached only 167 TPS, while maintaining a low latency of 0.04 seconds. Scalability analysis confirms its efficiency under increasing workloads, and IPFS integration enhances privacy and storage management. Security evaluations highlight ACHealthChain’s resilience against common threats, reinforcing its robustness. A key limitation of ACHealthChain is its reliance on Hyperledger Fabric, which operates within a permissioned network. While this enhances security, control, and privacy compliance, it may limit interoperability with public blockchain-based healthcare solutions that prioritize open and decentralized access. To address this, future work will focus on extending ACHealthChain to enhance interoperability with existing public healthcare infrastructures to enable seamless data exchange, support collaborative healthcare data analysis for improved decision-making, and integrate artificial intelligence to optimize access control policies and enhance security mechanisms, paving the way for advanced healthcare data management solutions.

## Data Availability

The framework code generated in this study is available in the GitHub repository (https://github.com/AhmedTawfik32/ACHealthChain_Framework).
